# Development and application of a UHPLC–MS/MS metabolomics based comprehensive systemic and tissue-specific screening method for inflammatory, oxidative and nitrosative stress

**DOI:** 10.1007/s00216-018-0912-2

**Published:** 2018-03-02

**Authors:** Johannes C. Schoeman, Amy C. Harms, Michel van Weeghel, Ruud Berger, Rob J. Vreeken, Thomas Hankemeier

**Affiliations:** 1 Department of Analytical Biosciences, Leiden Academic Centre for Drug Research, Leiden University, Einsteinweg 55, 2333 CC Leiden, Netherlands; 2 Netherlands Metabolomics Centre, Leiden University, Einsteinweg 55, 2333 CC Leiden, Netherlands; 3 Laboratory for Neurophysiology, Department of Molecular Cell Biology, Leiden University Medical Center, Einthovenweg 20, 2333 ZC Leiden, Netherlands; 4 Present Address: Department of Clinical Chemistry, Laboratory Genetic Metabolic Diseases, Academic Medical Center, University of Amsterdam, Meibergdreef 9, 1105 AZ Amsterdam, Netherlands; 5 Present Address: Discovery Sciences, Janssen R&D, Turnhoutseweg 30, 2340 Beerse, Belgium

**Keywords:** Metabolomics, Oxidative stress, Inflammation, Nitrosative stress, Liquid chromatography–tandem mass spectrometry

## Abstract

Oxidative stress and inflammation are underlying pathogenic mechanisms associated with the progression of several pathological conditions and immunological responses. Elucidating the role of signalling lipid classes, which include, among others, the isoprostanes, nitro fatty acids, prostanoids, sphingoid bases and lysophosphatidic acids, will create a snapshot of the cause and effect of inflammation and oxidative stress at the metabolic level. Here we describe a fast, sensitive, and targeted ultra-high-performance liquid chromatography–tandem mass spectrometry metabolomics method that allows the quantitative measurement and biological elucidation of 17 isoprostanes as well as their respective isomeric prostanoid mediators, three nitro fatty acids, four sphingoid mediators, and 24 lysophosphatidic acid species from serum as well as organ tissues, including liver, lung, heart, spleen, kidney and brain. Application of this method to paired mouse serum and tissue samples revealed tissue- and serum-specific stress and inflammatory readouts. Little correlation was found between localized (tissue) metabolite levels compared with the systemic (serum) circulation in a homeostatic model. The application of this method in future studies will enable us to explore the role of signalling lipids in the metabolic pathogenicity of stress and inflammation during health and disease.

**Electronic supplementary material:**

The online version of this article (10.1007/s00216-018-0912-2) contains supplementary material, which is available to authorized users.

## Introduction

**Fig. 1:**
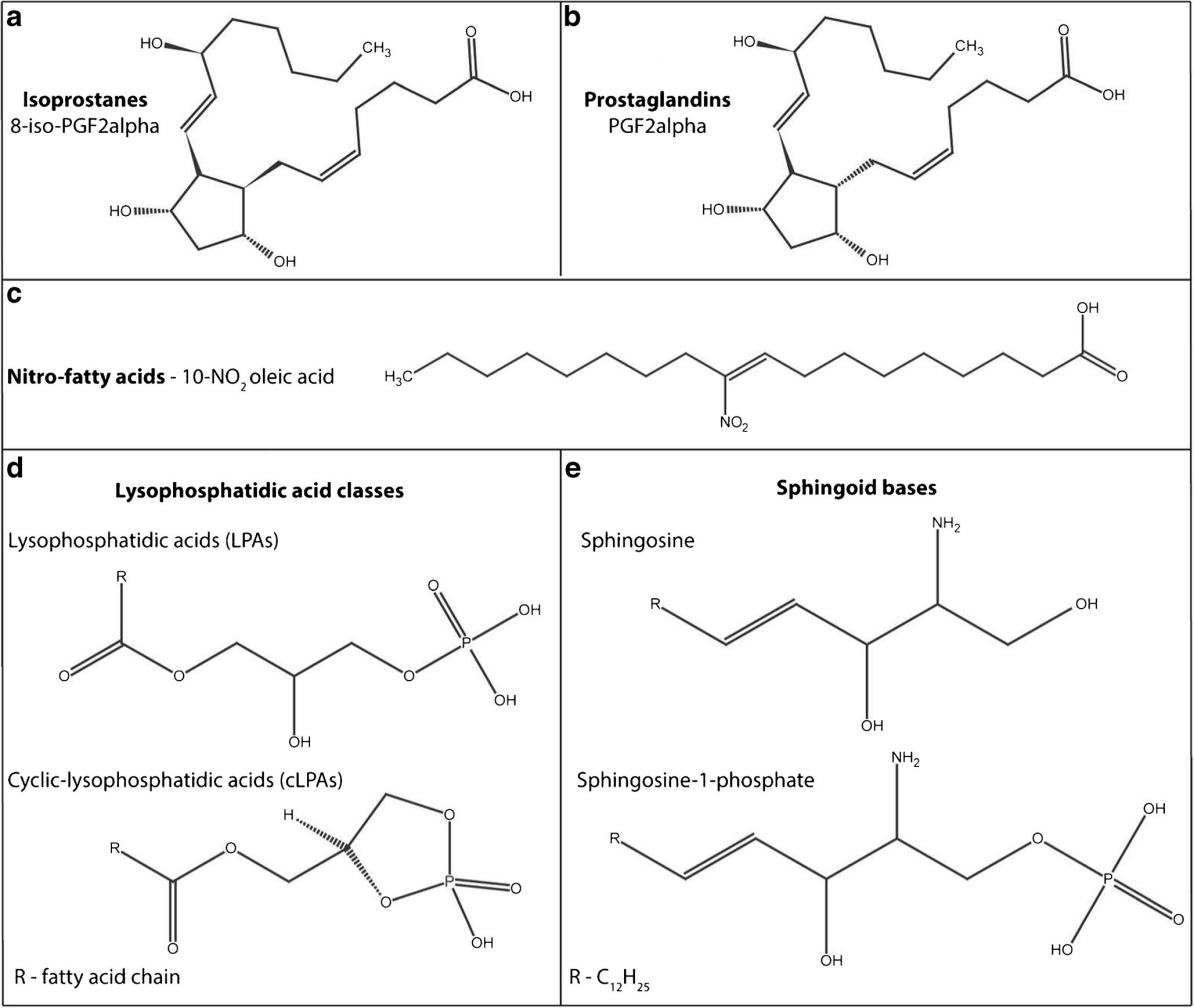
Structural overview of the signalling lipids constituting the panel of inflammatory, oxidative and nitrosative stress markers: **a** isoprostanes, **b** prostaglandins, **c**, nitro fatty acids, **d** lysophosphatidic acid classes and **e** sphingoid bases

Oxidative stress is characterized as a condition where the levels of reactive oxygen species (ROS) necessary for cellular redox biology [1–5] increase above the cellular antioxidant threshold, leading to macromolecular damage [6], and is an underlying pathogenic mechanism associated with the progression of most diseases [7–10]. The need to understand the intricate (cause-and-effect) relationship between oxidative stress and inflammation has been gaining momentum in recent years, as elucidating these mechanisms may allow novel therapeutic approaches. Because of the cumbrousness and unreliability in measuring free radicals, downstream products that are reflective of a failed cellular antioxidant capacity leading to oxidative damage are ideal metabolomics targets to evaluate oxidative stress. For example, the ratio of the reduced and oxidized glutathione species can be used as an oxidative stress readout [11]. The biological membrane bound glycerophospholipids are reservoirs for unsaturated fatty acids, vulnerable to free radical attacks [12, 13]. Non-enzymatic oxidation of these unsaturated fatty acids affects and impairs membrane integrity and function, leading to cellular stress. Isoprostanes (Fig. 1a) are stable prostanoid-like lipid peroxidation markers, with their readout regarded as the golden standard to evaluate oxidative stress in vitro and in vivo [14, 15]. Similarly, nitro fatty acids (NO_2_-FAs; Fig. 1c) synthesized via reactive nitrogen species (RNS) can be used to evaluate nitrosative stress within a system [16]. Although the downstream readouts of oxidative and nitrosative stress are effective for indirectly measuring the dysregulation in the ROS and RNS levels, measurement of lipid signalling metabolites implicated as causative of inflammation is also of importance. During innate immune activation via pathogen recognition receptors, the generation of ROS via the mitochondria and NADPH oxidases, and their downstream signalling, is essential for the activation of inflammatory pathways [17, 18].

Several lipid classes, including the prostaglandins, sphingoid bases and lysophosphatidic acids (LPAs) have been implicated in the activation of signalling pathways regulating inflammation, oxidative stress and cell proliferation among other physiological responses via dedicated cellular receptors. Prostaglandins (Fig. 1b) are enzymatic lipid signalling mediators playing an active role in inflammation, pain and immunomodulation, conducting their signalling through dedicated prostaglandin-specific G protein-coupled cellular receptors [19, 20]. Prostaglandins are enzymatically synthesized (de novo) via the cyclooxygenase-mediated oxidation of essential fatty acids. They are also structural isomers of the aforementioned isoprostanes. LPAs (Fig. 1d) are the simplest phospholipid species, an essential intermediate in de novo glycerophospholipid and triglyceride synthesis. Apart from their metabolic roles, LPAs and cyclic LPAs (cLPAs) are also active signalling mediators which are able to influence cell proliferation, immunological functions and inflammatory signalling through five dedicated G protein-coupled cellular receptors [21, 22]. The sphingoid bases (Fig. 1e) include the metabolites sphingosine and sphingosine 1-phosphate (S1P). The various roles of S1P in innate and adaptive immunity include immune cell trafficking, differentiation and immune surveillance [23–25], and S1P has also been implicated in inflammatory and oxidative stress signalling [26]. Development of a targeted metabolomics method for the measurements of isoprostanes, NO_2_-FAs, prostaglandins, sphingoids and LPAs as well as the cLPA species presents an opportunity to study the cause and effect of oxidative stress, inflammation and cell proliferation at the metabolic level. Secondly, studying the combined effect of these signalling mediators will be particularly helpful as these signalling molecules could have distinct and sometimes even opposing functions.

Several different methods have been developed for the analyses of these metabolites, each focusing on specific compound classes and biological matrixes. The analyses of prostanoids and isoprostanes is commonly accomplished with large volumes of plasma/serum (more than 200 μL), accompanied by solid-phase extraction coupled with liquid chromatography (LC)–tandem mass spectrometry (MS/MS) [27–30]. Comparatively, the analyses of lysophospholipids and sphingoid bases are facilitated by protein precipitation or liquid–liquid extraction (LLE) coupled with LC–MS/MS [31–35] with smaller volumes of plasma/serum (20–50 μL). Currently, no global signalling lipid metabolomics platform is available; this is because of the chemical diverseness and dynamic ranges of the signalling lipid classes. Here we describe a fast, sensitive and targeted ultra-high-performance LC (UHPLC)–MS/MS metabolomics method that allows the measurement of 17 isoprostanes as well as 11 isomeric prostaglandin mediators, three NO_2_-FAs, four sphingoid mediators, 16 LPAs and 6 cLPA species. Application of the method developed to patient-derived serum and tissue samples will help in determining the underlying links between inflammation and oxidative stress in disease. We applied the method developed to a homeostatic mouse model with paired tissue and serum samples, providing a stress and inflammation readout for serum as well as brain, lung, liver, heart, kidney and spleen tissues.

## Materials and methods

### Chemicals and reagents

Ultra-performance LC grade acetonitrile (ACN), isopropyl alcohol (IPA), methanol (MeOH), ethyl acetate (EtOAc) and water were purchased from Biosolve (Valkenswaard, Netherlands). 1-Butanol (BuOH) was obtained from Boom (Meppel, Netherlands). Acetic acid, ammonium hydroxide, butylated hydroxytoluene (BHT), ethylenediaminetetraacetic acid (EDTA) and ammonium acetate were from Sigma-Aldrich (Zwijndrecht, Netherlands). Sodium dihydrogen phosphate dehydrate and citric acid were obtained from Merck (Darmstadt, Germany). Standards and deuterated standards for the isoprostanes, prostanoids and NO_2_-FAs were purchased from Cayman Chemicals (Ann Arbor, MI, USA) (see Table S1). LPA and sphingoid base standards and uneven fatty acid length internal standards (ISTDs) were purchased from Avanti Polar Lipids (Alabaster, AL, USA) (Table S1).

### ISTDs and standard curve preparation

Standard and deuterated standard stock solutions were prepared in MeOH containing BHT (0.2 mg/mL). A calibration stock solution was made, with a concentration of 1304 nM for the isoprostanes, prostanoids and NO_2_-FAs and 4000 nM for the LPA and sphingoid base standards and was labelled “C8”. This C8 solution was diluted to levels C7 to C1, with C1 being the lowest calibration level at 0.75 and 2.3 nM, respectively. From these calibration stock mixes (C8 to C1), 20 μL was added to 150 μL sample to construct the calibration curves. Table S2 provides a schematic overview of the stock solution concentrations as well as the spiked calibration concentrations used during this study and method validation procedures.

### Biological samples

#### Human serum

Control human serum used in the method validation was purchased from Harlan Sera-Lab (Leicester, UK; tested negative for HIV antibody and hepatitis B surface antigen).

#### C57BL/6 mouse sample collection

C57BL/6 mice were housed at 21 ± 1 °C, 40–50% humidity, with a 12-h light-dark cycle, with ad libitum access to water and a standard rodent diet. Eight male adult mice were anesthetized with isoflurane and euthanized by cervical dislocation. After the chest cavity had been opened, blood was collected with a 22-gauge needle from the heart and left to coagulate on ice. The heart, lungs, liver, spleen, kidneys and brain were harvested in that sequence, cleaned of excess visceral fat, hair, tissues and blood, and snap frozen in liquid nitrogen. After the blood had coagulated on ice for approximately 30 min, the samples were centrifuged at 2000*g* at 4 °C for 10 min, after which the serum was transferred to a clean Eppendorf vial, snap frozen and stored at –80 °C. All procedures were approved by the Institutional Review Board for Animal Experiments at Leiden University Medical Center (Leiden, Netherlands).

### Extraction procedures

#### Serum extraction

Serum aliquots (150 μL) were thawed on ice, after which 5 μL antioxidant (0.2mg BHT and 0.2 mg EDTA) solution and 10 μL of ISTD solution were added and the serum was briefly vortexed. Samples were then acidified through the addition of 350 uL of 0.2 M citric acid and 0.1 M disodium hydrogen phosphate buffer at pH 4.5. LLE was accomplished by the addition of 1 mL of a 1-butanol–ethyl acetate (1:1 v/v) solution. Samples were mixed for 2 min, then centrifuged for 10 min at 4 °C and 25,300*g*, after which 900 μL of the upper organic phase was collected and transferred to a clean 2-mL tube. The LLE was repeated by addition of 400 μL BuOH saturated with water and 400 μL EtOAc to the remaining aqueous phase. After mixing and centrifugation, 800 μL of the organic phase was collected, and the total organic fraction was dried under a vacuum. Then 40 μL of ice-cold 90% MeOH injection solution was added to the dried residue as a reconstitution solution. Reconstituted samples were vortexed before centrifugation for 10 min at 4 °C and 25,300*g*, and were subsequently transferred to inserts in injection vials before LC–mass spectrometry (MS) analyses and stored in the autosampler at 5 °C.

#### Tissue extraction

Snap-frozen tissue samples were transferred to a freeze dryer, where tissues were dried overnight, mechanically homogenized, aliquoted and stored at –80 °C before extraction. Dried tissue amounts of 5 mg were suspended in 200 μL of a 0.2 M citric acid and 0.1 M disodium hydrogen phosphate buffer at pH 4.5, to which approximately 500 mg of 0.5-mm stainless steel beads (Next Advance, Averill Park, NY, USA) was added. Then 5 μL antioxidant (0.2mg BHT and 0.2 mg EDTA) and 10 μL ISTD solution were added, and tissue samples were homogenized in a bullet blender at maximum speed for 10 min. Afterwards, samples were centrifuged for 30 s at 2325*g*, followed by the addition of 150 μL of pH 4.5 buffer as well as 1 mL of a BuOH–EtOAc (1:1 v/v) solution. The collection of the organic phase and the repeated extraction steps were as detailed in the previous subsection for serum extraction.

### Targeted LC–MS/MS analyses

#### Low-pH runs

LC was performed with an LCMS-8050 system (Shimadzu, Japan) and an Acquity BEH C_18_ column (50 mm × 2.1 mm, 1.7 μm; Waters, Milford, MA, USA) maintained at 40 °C. The mobile phases consisted of water and 0.1% acetic acid (mobile phase A), ACN–MeOH (7.5:2.5 v/v) and 0.1% acetic acid (mobile phase B), and IPA (mobile phase C) with a flow rate of 0.7 mL/min. The pH of the mobile phases ranged between 3.2 and 3.5 during the chromatographic gradient. To increase the column load ability, a stacked injection was used which consisted of stacking 10-μL sample volume with 20 μL of mobile phase A in the needle before injection of this “solution stack” onto the column. The analytes were eluted with a gradient starting at 5% mobile phase B and 0% mobile phase C an progressing to 75% mobile phase B and 25% mobile phase C between 0 and 9 min; the final conditions were held for 1 min, after which the column was reequilibrated to the starting conditions from 10.15 to 13 min.

#### High-pH runs

LC was performed with an LCMS-8050 system (Shimadzu, Japan) and a Kromasil EternityXT-1.8 C_18_ column (50 mm × 2.1 mm, 1.8 μm; AkzoNobel, Netherlands) maintained at 40 °C. The mobile phases consisted of water, 5 mM ammonium acetate and 0.0625% ammonium hydroxide (mobile phase A) and ACN–IPA (8:2 v/v) and 0.1% ammonium hydroxide (mobile phase B) with a flow rate of 0.6 mL/min. The pH of the mobile phases ranged between 10.3 and 8.5 during the chromatographic gradient. The injection volume was 5 μL. The metabolites were eluted with a linear gradient starting at 10% mobile phase B and progressing to 100% mobile phase B in 5 min; the conditions were kept at 100% mobile phase B for 0.75 min, after which the column was reequilibrated to the starting conditions from 5.75 to 8 min.

#### MS/MS analyses

The Shimadzu LCMS-8050 system consists of a UHPLC system connected to a triple-quadrupole mass spectrometer with an electrospray ionization source. The electrospray ionization source parameters were as follows: interface temperature 300 °C, desolvation line temperature 250 °C, heat block temperature 400 °C, nebulizing gas flow rate 3 L/min, heating gas flow rate 10 L/min and drying gas flow rate 10 L/min. The analytes and ISTDs were measured by multiple reaction monitoring (MRM) in either positive or negative ion mode with the complete optimized target list (collision energy and dwell time) in Table S1. During the development procedure, the most specific or sensitive transition was selected to avoid interferences and increase detection limits.

The target list (Table S1) included both metabolites identified with use of commercially available standards and putatively identified metabolites. The putatively identified metabolites were identified with use of different MS modes and expected transitions, as explained next.

Because of the shortage of LPA standards, an MS screening approach was used to putatively identify other LPA species. Precursor ion scans together with single reaction monitoring (SRM) was used to search for and identify putative LPA species with use of known fragmentation patterns. Metabolites were identified on the basis of the following criteria:

1. The retention times of putatively identified metabolites were compared with those standards and had to have similar retention time frames.
2. Elution sequences were compared with those of the available standards. For example, the most unsaturated acyl species of any given length was eluted first, followed by progressively more saturated species until the saturated one (LPA C18:3 will be eluted first, followed by LPA C18:2, LPA C18:1 and lastly LPA C18:0).
3. The putatively identified metabolites had to have the characteristic fragmentation patterns associated with the LPA and cLPA species. For the LPA species the deprotonated parent ion [M − H]− had to fragment into a dehydrated glycerol phosphate 152.90 m/z fragment as well as a phosphate 78.90 m/z fragment. For the cLPA species the deprotonated parent ion [M – H]− had to fragment into a unique free fatty acid fragment as well as a phosphate 78.90 m/z fragment.

The targeted LPA species are listed in Table S3, with an overview of the single reaction monitoring transitions and fragmentation patterns. After identification, MRM transitions of putatively identified metabolites were included in the method using the class-representative commercial standards in optimizing their MS parameters.

### Method validation criteria

Method performance was investigated, and included linearity, limits of detection, retention time stability, interday and intraday precision, extraction recovery and the matrix effect.

#### Limit of detection and linear range

Calibrations curves were prepared in four replicates with seven concentrations ranging over four orders of magnitude to assess the limit of detection (with use of a blank matrix consisting of pure water) and linear range (with use of control serum). For each calibration curve, the ratio of the analyte area and its corresponding ISTD area was plotted against its nominal concentration, with no weighting factor being applied. The limit of detection was determined as the lowest concentration that resulted in a peak with a signal-to-noise ratio greater than 3 according to the ASTM method. Furthermore, the lower limit of quantification used to determine the linear range was determined as the lowest concentration that resulted in a peak with a signal-to-noise ratio greater than 10 according to the ASTM method.

#### Retention time and autosampler stability

Retention time stability reflects the stability of the chromatography over progressive runs, to ensure correct peak identification based on retention time during data processing. The retention time stability was investigated through our determining the relative standard deviations (RSD) of the metabolite retention time across a sequence of 100 injections. Retention times are considered stabile if the RSD is less than 1%. The autosampler performance was investigated through our determining the RSD of the peak areas of 12-[(cyclohexylcarbamoyl)amino]dodecanoic acid (CUDA). CUDA is an exogenous compound added to the injection solution at a level of 100 nM. Autosampler performance was deemed to be adequate when the CUDA peak areas had an RSD of less than 5% across a batch.

#### Intraday and interday precision

Precision is defined as the closeness of the measurements of individual samples when the procedure is applied to multiple aliquots of a homogenous matrix; hence it is reported as the RSD of the measurements [36]. The method is considered to be precise if the RSD is below 15% for mid calibration range metabolites or 30% for lower limit of detection range metabolites. Intraday precision was determined by our calculating the RSD of four replicate measurements of control serum at three different levels (low, medium and high; Table S2). Interday precision was assessed by our comparing the closeness of the quadruplicate samples at each level over 3 days.

#### Analytical recovery

Recovery reflects the extraction efficiency of the LLE procedure for the metabolites in a specific biological matrix and should be reproducible (low RSD). Both the serum and different tissue recoveries were determined by our comparing the response of an ISTD added to the sample before LLE with the response obtained from the ISTD added after LLE. Serum recoveries were determined in quadruplicate with control serum aliquots at three different levels (low, medium, high; Table S2). Tissue recoveries were determined in quadruplicate with control tissue aliquots at one level (medium; Table S2).

#### Matrix effect

The matrix effect can be explained as the interference of matrix compounds during sample preparation and interferences in ionization efficiency resulting from co-eluted compounds present in the biological matrix, and must be evaluated [37]. If a matrix effect is affecting the analyte signal, this does not necessarily imply that the method is not valid, but in this case, the added ISTD must be able to correct for the matrix effects. The matrix effect was assessed by our adding the non-endogenous ISTD to the matrix and to an academic blank solution (pure water), and by our comparing the ISTD responses (i.e. peak areas without further corrections) obtained from the spiked blank and the ISTD responses obtained from the biological matrix spiked with the ISTD before LLE. For serum, the matrix effect was determined in quadruplicate from control serum and blank matrix aliquots at three different ISTD levels (low, medium and high; Table S2). For tissues, the matrix effect was determined in quadruplicate from control tissues and control blank solution aliquots at a single ISTD level (medium; Table S2).

### Data processing and statistical methods

Peak detection, integration and quantification were done with the Shimadzu LabSolution software package (version 5.65). The relative ratios of metabolite peak areas to the peak areas of their corresponding ISTDs were used for statistical analyses. Principal component analyses (PCA) and heatmaps based on Euclidean distance measure and the Ward clustering algorithm were performed with the R script-based online tool MetaboAnalyst (version 3.0), a comprehensive tool suitable for analysing metabolomics data [38]. Spearman correlation coefficients were calculated with IBM SPSS Statistics version 23.0 (IBM, Armonk, NY, USA). During the intergroup correlation analyses, each metabolite was correlated against itself; thus, no multiple testing correction was performed. Significant correlations were defined as a Spearman coefficient *r* less than -0.7 or greater than 0.7 and *p* < 0.05.

## Results and discussion

### Method development

The development of a high-throughput method for the quantification of a panel of inflammatory, oxidative and nitrosative stress markers in serum (systemic readout) and tissues (localized readout) faced five main challenges. Firstly, the dynamic range of endogenous concentrations, ranging from the low nanomolar range for the isoprostanes, prostaglandins and NO_2_-FAs [39, 40] to medium to high nanomolar ranges for the LPAs and sphingoids [41, 42], demanded optimization of every step to guarantee the optimal limits of quantification. Because of the diverse chemical nature of these metabolites, LLE was chosen as the sample preparation method. The pH 4.5 buffer was used to ensure stability and a negative charge of the target metabolites. In the presence of strong acids, the values for LPAs obtained in an analysis can be artificially increased ex vivo by either enzymatic or chemical hydrolysis of lysophosphatidylcholines and lysophosphatidylethanolamines to form LPA [42, 43]. The composition of BuOH and EtOAc used during LLE ensured excellent recoveries of our target LPAs, cLPAs and sphingoids [32, 42] as well as oxidized and nitrated lipids. The polar nature of the organic solvents used during LLE also reduced background noise and matrix interferences by preventing the extraction of non-polar and neutral lipids.

**Fig. 2:**
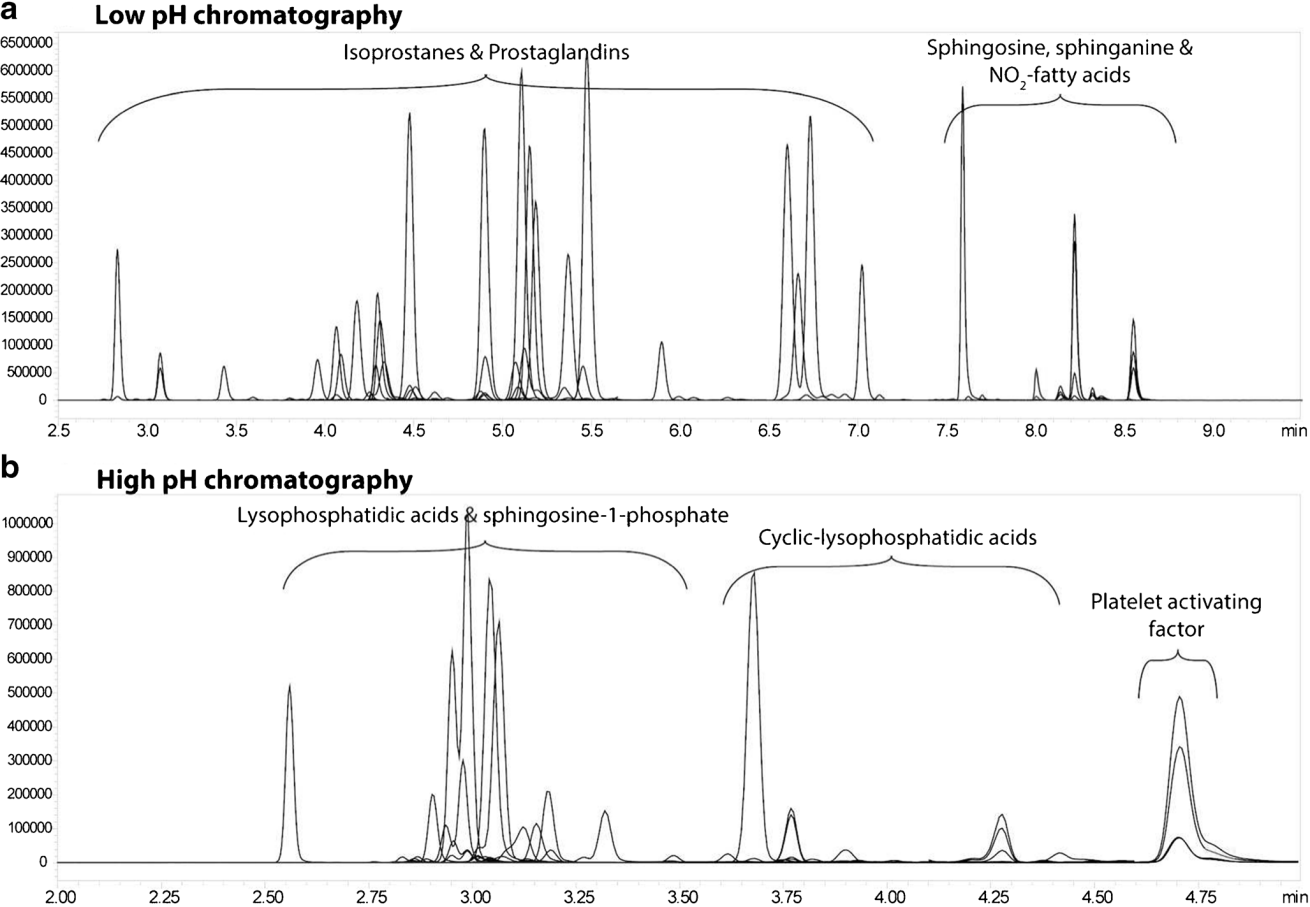
Liquid chromatography–tandem mass spectrometry chromatograms from **a** the low-pH run and **b** the high-pH run

Secondly, optimized, MS-compatible chromatography is necessary for adequate metabolite separation which can distinguish between the structural isomers of isoprostanes and prostaglandins (Fig. S1). Furthermore, separation between different lysophospholipid classes is also necessary for accurate measurements of LPA levels, as in-source fragmentation of more complex lysophospholipids (lysophosphatidylserine, lysophosphatidylinositol, lysophosphatidylglycerol, lysophosphatidylcholine, lysophosphatidylethanolamine) can result in increased LPA levels in the case of chromatographic co-elution (Fig. S2). Optimizing the chromatographic run proved the most challenging aspect and required compromises in (1) mobile phase conditions, including organic solvents, salt concentrations and pH modifiers, (2) gradients and run time and (3) different C_18_ stationary phases; no single chromatographic run was found to be suitable for the analysis of the selected panel of compounds. Table S4 provides an overview of the conditions tested and each class’s response in the selected setup. To be able to analyse all compound classes, the chromatography was split into two runs; namely (1) a low-pH run analysing the isoprostanes, prostaglandins and NO_2_-FAs as well as two sphingoids (sphingosine and sphinganine) (Fig. 2a) and (2) a high-pH run analysing the LPA classes as well as the two phosphate sphingoids S1P and sphinganine 1-phosphate (Fig. 2b).

Thirdly, optimization of MS detection is necessary to ensure accurate quantification of the endogenous metabolites over a large dynamic range. An MS/MS approach using dynamic MRM was chosen for enhanced sensitivity. MS parameters were individually optimized for each compound with use of commercially available standards. The MS-optimized compounds and their respective deuterated ISTDs were evaluated for MRM crosstalk, and no interferences were observed. Possible crosstalk between MRM transitions was further reduced by use of a 2-ms pause time between each MRM transition so that ions of the previous transition did not interfere. The fast polarity switching of 5 ms of the LCMS-8050 system and optimized chromatography ensured no drop in sensitivity when measurements were made in both the negative MS mode and the positive MS mode during chromatographic runs.

Fourthly, ISTDs are necessary to compensate for (1) variation in extraction efficiency during LLE, (2) instrument variability and (3) ionization efficiency. When possible, deuterium-labelled standards, usually containing 4 to 11 deuterium atoms, were used as the ISTD to ensure MRM discrimination from the endogenous metabolites. These labelled ISTDs have very similar properties in terms of extraction, recovery and elution compared with the endogenous unlabelled metabolite. Because of the limited availability of deuterated LPAs and sphingoids, uneven chain fatty acid (C17:0) species were used as ISTDs for the quantification of endogenous LPAs, cLPAs and sphingoids. When no direct/deuterated ISTD was available for a metabolite, the ISTD that was eluted closest to that endogenous metabolite of the same class was selected as the ISTD.

Lastly, measuring a localized response requires the use of tissue as the biological matrix. As different tissues have vastly different functions, it is presumed that each tissue will have a unique homeostatic profile of stress and inflammatory mediators. Tissue sampling also presented some matrix-specific challenges, and the choice between using wet or dry material is not always clear and could influence extraction repeatability and efficiency. In this study, we used freeze-dried tissues, which were mechanically homogenized, leading to more reproducible sample aliquots. Tissue samples were subsequently further homogenized during the LLE in the extraction buffer, with use of 0.5-mm stainless steel beads and a bullet blender.

### Method validation

The targeted metabolomics profiling platform was validated to ensure its robustness and reproducibility in providing quality data for biological interpretation. As the selected target metabolites are mostly present in biological matrices, including serum and tissues, no “blank” matrix was available, and different validation matrices had to be used for different experiments [36, 44]. Thus, for calibration lines investigating detector linearity, a blank matrix of pure water was selected. For investigation of intraday and interday precision, homogeneous biological matrix aliquots (commercially available serum) as well as a blank matrix (water) were spiked with the panel of compounds. Recoveries and matrix effects were studied with only exogenous ISTDs added to homogeneous biological matrix aliquots.

#### Serum validation

Investigation of the linearity and sensitivity of our metabolomics platform with the LCMS-8050 system provided satisfactory results. Overall, the 40 standards representative of the different endogenous compound classes in the targeted metabolomics platform showed a good linear response, with 87.5% of the metabolites having *R*^2^ >0.99 and the remaining 12.5% having 0.96 < *R*^2^ <0.99 (Table 1). Regarding sensitivity, the limit of detection was determined as the lowest amount of standard necessary to provide an signal-to-noise ratio greater than 3 while still being close to the linear range of the calibration curve (Table 1). The linear ranges for the different compound classes were comparable to known referenced human physiological levels for the most characterized compounds [41, 45, 46]. For example, the concentrations of prostaglandin E_2_ (PGE_2_) and the isoprostane 8-isoprostaglandin F_2α_ (8-iso-PGF_2α_) ranged from 0.5 to 1 nM and from 0.25 to 0.6 nM, respectively, in healthy patients [45]. Total LPA and S1P plasma concentrations have been reported to range from 0.14 to 1.64 μM and from 352.7 to 413.72 nM, respectively, in healthy individuals [41, 46]. The prostaglandins and isoprostanes had limits of detection of approximately 0.09 nM, corresponding to a limit of detection in serum between 20 and 40 pg/mL for the different metabolites. The limits of detection for LPAs, cLPAs and sphingoids were higher than those for the other classes because of increased background noise but were still below physiological levels.

**Table 1:** Method validation characteristics in serum

Compound	Linearity^a^ (*R*^2^)	LOD (nM)	Linear range (nM)	RT stability (*n* = 3)^b^ (RSD, %)	Precision in serum (RSD, %)					
					Intraday			Interday		
					C2–low	C4–medium	C7–high	C2–low	C4–medium	C7–high
Lysophosphatidic acid classes										
aLPA C18:1	0.9963	1	4-533	0.79	19.9	8.8	27.3	11.5	8.5	30.1
cLPA C18:1	0.9972	1	4-533	1.41	10.7	11.2	16.4	26.0	12.9	12.5
LPA C20:4	0.9914	4	42-533	1.81	7.9	13.3	33.9	26.8	17.9	23.9
LPA C16:0	0.9931	1	4-533	1.88	2.9	14.4	7.7	26.2	11.6	25.3
LPA C18:0	0.9899	1	4-533	2.25	26.7	12.2	44.7	26.8	12.9	34.6
Sphingoids										
Sph C18:1	0.9942	1	4-533	0.13	0.3	4.8	4.9	7.5	8.2	11
Spha C18:0	0.9966	1	4-533	0.28	21	3.3	18.8	13.6	9.8	16.7
S1P C18:1	0.997	1	4-533	1.52	4.9	9.3	24.0	8.4	16.2	21.0
Nitro fatty acids										
NO_2_-OA	0.9995	0.09	0.3-173	0.04	9.9	5.7	9.1	9.2	6.0	10.4
NO_2_-LA	0.9993	0.09	0.3-173	0.04	16.3	8.5	9.5	14.9	17.5	20.7
Prostaglandins										
2,3-Dinor-11b-PGF_2α_	0.9998	0.09	0.3-173	0.21	20.8	8.3	9.8	13.8	6.6	5.5
PGE_3_	0.9989	0.09	0.3-173	0.26	12.2	1.6	18.9	7.0	9.2	12.6
PGD_3_	0.9996	0.09	0.3-173	0.21	7.2	8.8	15.2	7.7	6.6	7.7
PGF_3α_	0.9998	0.3	1 -173	0.17	17.2	5.4	4.9	17.0	6.6	7.1
PGE_2_	0.9999	0.09	0.3-173	0.13	6.3	2.0	9.6	11.6	11.2	11.1
PGE_1_	0.9996	0.3	1 -173	0.10	1.5	3.4	6.9	5.6	5.0	3.9
PGD_2_	0.9991	0.09	0.3-173	0.20	1.1	2.6	1.5	1.2	2.3	1.6
PGF_1α_	0.9995	0.09	0.3-173	0.09	29.8	13.1	18.8	89.0	15.3	12.2
PGF_2α_	0.9987	0.09	0.3-173	0.09	15.3	6.6	6.2	8.8	5.1	5.2
13,14-Dihydro- PGF_2α_	0.9995	0.09	0.3-173	0.20	2.6	4.7	5.2	2.9	3.4	3.7
PGA_2_	0.9994	0.09	0.3-173	0.10	7.6	0.6	6.1	6.0	4.0	4.7
PGA_1_	0.9995	0.09	0.3-173	0.07	1.9	0.7	5.8	2.7	7.1	5.5
Isoprostanes										
2,3-Dinor-8-iso-PGF_2α_	0.9995	0.09	0.3-173	0.25	9.5	2.7	3.6	11.4	4.3	3.9
8-Iso-PGF_3α_	0.9995	0.3	1 -173	0.16	3.5	2.1	2.8	150.0	4.5	8.0
8-Iso-15-keto-PGF_2β_	0.9982	0.09	0.3-173	0.20	9.1	5.3	9.2	12.2	5.8	7.5
8-Iso-15-keto-PGE_2_	0.9997	1.3	5-173	0.55	38.3	2.7	6.9	24.9	2.7	3.6
8-Iso-15-keto-PGF_2α_	0.9976	0.09	0.3-173	0.13	2.6	4.0	2.3	15.9	8.1	11.6
iPF_2α_	0.9988	0.09	0.3-173	0.12	8.5	4.2	6.8	5.5	2.7	4.2
8-Iso-(15*R*)-PGF_2α_	0.9991	0.09	0.3-173	0.12	10.4	10.2	9.7	16.3	12.1	13.8
8-Iso-PGF_1α_	0.9989	0.09	0.3-173	0.23	1.1	4.0	3.9	3.7	4.1	3.2
8-Iso-13,14-dihydro-PGF_2α_	0.9994	0.3	1 -173	0.11	10.6	3.7	6.4	150.8	8.8	11.6
8-Iso-PGF_2α_	0.9993	0.09	0.3-173	0.11	8.1	5.1	5.3	9.3	5.1	6.2
8-Iso-PGE_2_	0.9997	0.09	0.3-173	0.12	5.9	4.2	2.2	10.9	19.2	1.7
8-Iso-PGE_1_	0.9995	0.09	0.3-173	0.15	8.2	3.8	4.8	43.6	6.9	3.9
5iPF2α	0.9998	0.09	0.3-173	0.10	5.8	2.5	5.2	6.2	5.9	7.2
8-Iso-PGA_2_	0.9994	0.09	0.3-173	0.11	4.9	1.5	5.8	5.9	4.1	4.8
8-Iso-PGA_1_	0.9993	0.09	0.3-173	0.42	27.4	9.3	5.7	21.7	7.6	5.9
8,12-iPF^2α^ IV	0.9974	0.09	0.3-173	0.08	7.0	4.6	9.9	4.1	5.2	16.0

*aLPA* alkyl lysophosphatidic acid, *cLPA* cyclic lysophosphatidic acid, *iP* isoprostane, *LA* linoleic acid, *LOD* limit of detection, *LPA* lysophosphatidic acid, *OA* oleic acid, *PG* prostaglandin, *RSD* relative standard deviation, *RT* retention time, *Sph* sphingosine, *Spha* sphinganine, *S1P* sphingosine 1-phosphate

^a^Calibration curves for all standards can be found in Fig. S3.

^b^RT stability was calculated over three separate batches on three separate days.

Retention time stability was investigated for both the low-pH run compounds and the high-pH run compounds across three different batches measured on three separate days. Retention time stability is critical to ensure correct peak identification over different sample batches, as a number of structural isomers of the prostaglandins and isoprostanes have to be differentiated, and co-eluted lysophospholipids can influence the response of LPAs because of in-source fragmentation. The low-pH chromatography performed best with the highest RSD or the retention time being 0.55% for 8-iso-15-keto-PGE_2_ (Table 1). The high-pH chromatography had slightly higher RSDs for retention times, with LPA C18:0 showing an RSD of 2.25% (Table 1). The RSD of 2.25% corresponds to a standard deviation of 0.075 min, or 4.5 s, which still allows correct peak identification, although care should be taken during peak integration across batches. Likewise, the autosampler stability proved adequate, with the exogenous reconstitution compound CUDA having an RSD of less than 5% across a batch.

**Fig. 3:**
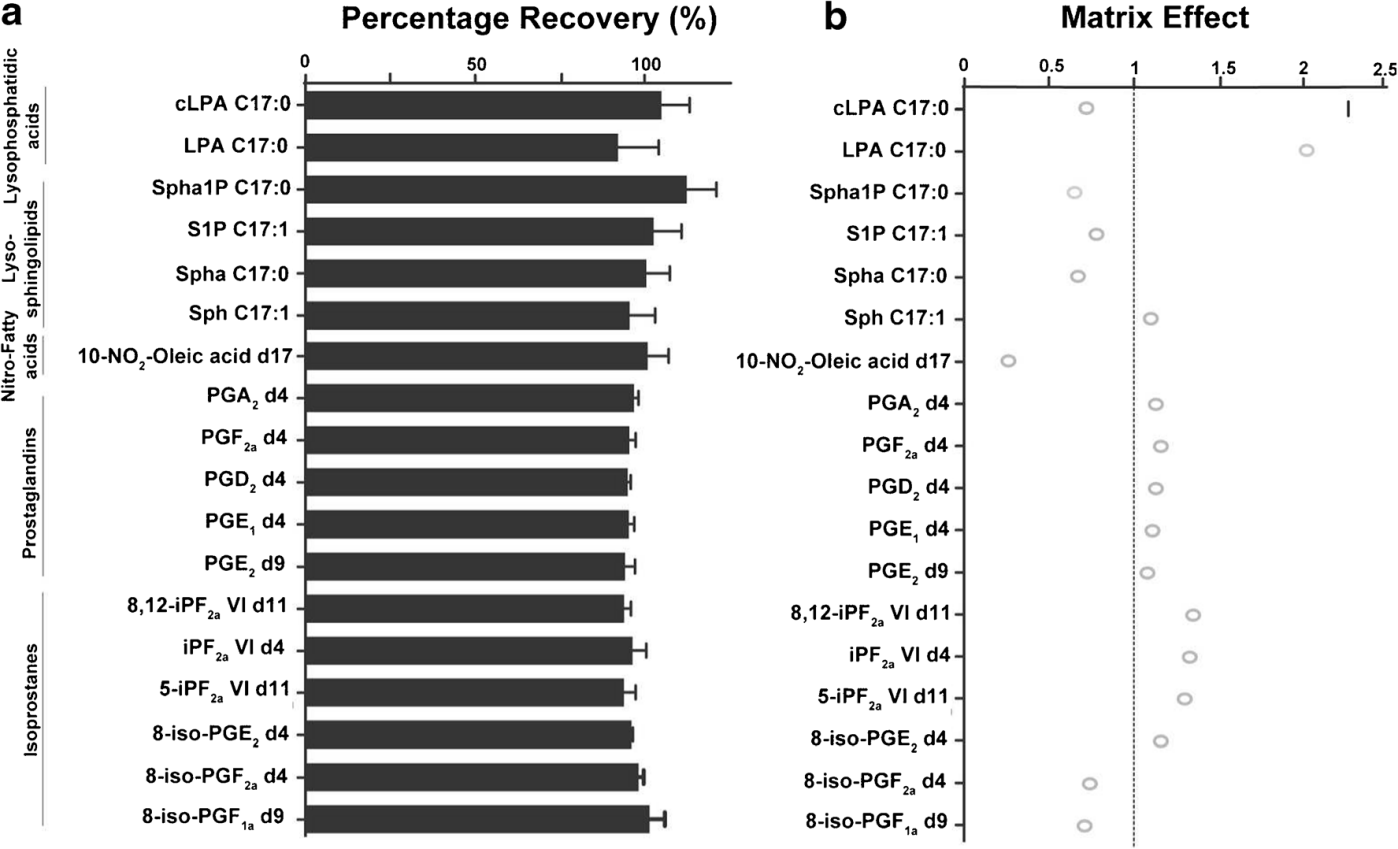
During serum method validation the internal standards were investigated for recovery (a) and the matrix effect (b), with 1 equal to no matrix effect. Error bars represent the relative standard deviations, with *n* = 4. cLPA cyclic lysophosphatidic acid, iP isoprostane, LPA lysophosphatidic acid, PG prostaglandin, S1P sphingosine 1-phosphate, Sph sphingosine, Spha sphinganine, Spha1P sphinganine 1-phosphate

The intraday precision was found to be good across the three levels investigated in a serum matrix, with 100% of metabolites having an RSD of less than 15% at the C4 level, and 73% having an RSD of less than 15% at the C2 level (Table 1). Next, we assessed the overall reproducibility considering variables such as extraction, measurement days and instrument-related issues, including injection variation or MS drift. Interday precision gave equally satisfactory results, with 77% of metabolites having an RSD of less than 15% and 94% having an RSD of les than 30% across the three levels investigated (Table 1). Overall the LPAs and sphingoids had higher RSDs, which is presumably due to the lack of deuterated ISTDs as then uneven (C17:0) acyl derivatives of these metabolites were used. Recovery experiments demonstrated minimal loss of metabolites during the LLE serum extraction procedure. Averaged ISTD recoveries over three consecutive days were close to 100% (Fig. 3, panel a, Table S5). Higher extraction variation was observed in the LPA, sphingoid and NO_2_-FAs ISTDs as compared with the isoprostane and prostaglandin ISTDs; the highest RSDs were still well below 15%, which also provides a further glimpse into the precision of the method. The matrix effect values (Fig. 3, panel b, Table S5) were close to 1 for most of the metabolites, an indication that the extraction method and the serum matrix have minimal impact during MS/MS detection for most of the targeted metabolites. The LPA C17:0 ISTD showed some ion enhancement, and especially the NO_2_-FAs experienced higher matrix effects than the other compounds.

#### Tissue application and performance characteristics

When questions related to health and disease are being addressed, serum provides a systemic readout for stress and inflammation markers. Measuring these metabolites in tissues, on the other hand, would provide a localized readout reflective of tissue homeostasis. Therefore, we also optimized the method developed for the extraction of heart, liver, lung, brain, spleen and kidney tissues and investigated the performance in each of these matrices. The performance characteristics evaluated for tissue samples included metabolite recoveries, matrix effects and intraday precision. We attempted to compensate for tissue heterogeneity (biological variation) by using pooled dried tissues. These pooled samples were tissue specific and consisted of mechanically homogenized dried tissues, which were subsequently aliquoted to represent replicates of “homogeneous biological tissues”. These homogeneous biological tissue replicates were then used to investigate intraday precision, and with the addition of ISTD, we could assess the recovery and matrix effect for each tissue independently.

**Fig. 4:**
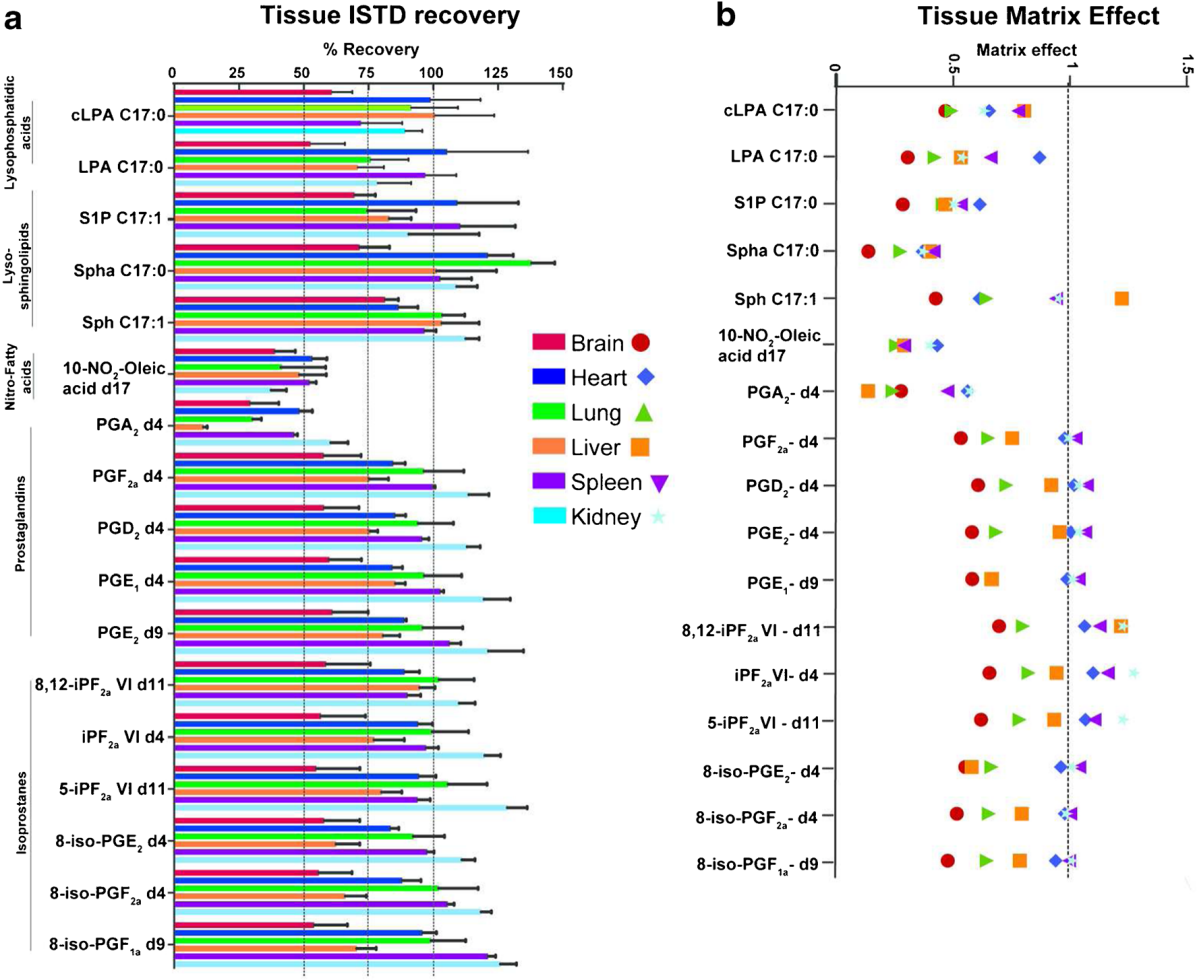
During the tissue method validation the internal standards were investigated for recovery (a) and the matrix effect (b) in brain, heart, liver, kidney, lung and spleen samples. Error bars represent the relative standard deviations, with *n* = 4. cLPA cyclic lysophosphatidic acid, iP isoprostane, LPA lysophosphatidic acid, PG prostaglandin, S1P sphingosine 1-phosphate, Sph sphingosine, Spha sphinganine, Spha1P sphinganine 1-phosphate

The different recoveries of the ISTDs from the six tissues reflected the diverse nature of these tissues (Fig. 4, panel a, Table S6). It is important to note that the recoveries reported here do not assess the extent of metabolite recovery from tissue (solid to liquid) but assess only the eventual loss of targeted metabolites during the LLE sample preparation. Overall, brain tissue had the lowest levels of recoveries across the whole panel of ISTDs. We attribute this finding to the composition of brain tissue, consisting predominantly of very long chain lipid species. The polar nature of the organic solvent prevented the extraction of non-polar lipid species; therefore, during the LLE, a white lipid interphase was formed between the buffer and organic solvent and this probably negatively impacted the extraction efficiency of the panel of lipid signalling mediators. On the other hand, kidney tissue had the highest metabolite recovery rates. The NO_2_-FA ISTD was poorly extracted, with recoveries ranging from 36% to 55% across all tissues. Blank matrix (H_2_O) samples following the same tissue extraction procedure showed an extraction efficiency of approximately 80%. Investigation of the reproducibility (*n* = 4) of 10-nitrooleate-*d*_17_ revealed RSDs ranging from 4% in spleen tissue to 30% in lung tissue. Hence although the NO_2_-FAs have poor extraction efficiencies, in some tissues they can be reproducibly measured. In addition, these results suggest interactions of NO_2_-FAs with tissue or protein during extraction, negatively impacting on the extraction efficacy of the NO_2_-FAs. The isoprostanes and prostaglandins [except for prostaglandin A2 (PGA_2_)-*d*_4_] had reasonable extraction efficiencies (70–100%) as did the LPA, cLPA and sphingoid metabolites.

From investigation of the matrix effect for different tissues (Fig. 4, panel b, Table S6), brain tissue, having the lowest recoveries, also experienced the highest levels of the matrix effect. Kidney tissue, with its high recoveries, together with spleen tissue, experienced the least matrix effects for most of the compounds. Compounds eluted later in the LC–MS chromatogram experienced notably higher levels of the matrix effect compared with the early eluted isoprostanes and prostaglandins. This could be attributed to the co-elution of other lipid species, most possibly other lysophospholipid metabolites present in high concentration in the tissues and extracted during the LLE step; and the levels of these lipid species might be higher in brain, lung, and liver compared with heart, kidney and spleen. Notably, NO_2_-FAs experienced high levels of the matrix effect during the LC–MS chromatographic run, and together with the reduced extraction efficiency of this class of metabolites, this highlights the challenges in measuring them. The use of ISTDs for the analysis of especially those metabolites showing higher matrix effects is therefore critical; these non-endogenous ISTDs should be added at the same concentration as the corresponding metabolite in different tissues and allow a better comparison across different tissues in biological studies, and this is true for semiquantitative (as in our case) as well as quantitative metabolomics measurements.

The intraday precision was determined for all commercially available endogenous metabolites added to the different pooled tissue samples (*n* = 4) at the C4 (medium) level (Fig. S4, Table S7). The intraday precision results showed the extraction method to be reproducible, with all six tissues showing around 90% of detected metabolites having an RSD of less than 30%. For extraction of comparable metabolites from muscle tissue Alves et al. [47] reported higher RSDs, but still found that individual sample (biological) variation was greater than replicate (procedure) variation. The precision was especially good for those metabolites for which deuterated ISTDs were available: both nitro-oleic acid and PGA_2_ had RSDs below 16% and 8%, respectively, across all six tissues (Table S7). Furthermore, from comparison of serum and tissue results, we can generate a tissue-specific stress and inflammatory readout in the tissue samples, contributing to fully understanding the localized responses of these metabolites in health and disease.

### Metabolic profiling of healthy paired mouse serum and tissue samples

Applying the method developed to paired tissue and serum samples from eight healthy C57BL/6 mice, we could create a homeostatic stress (oxidative and nitrosative) and inflammatory metabolic profile. Of the seven biological matrices investigated, spleen had the highest number of metabolites (53 of 60 metabolites) detected, and serum had the lowest number (35 of 60 metabolites). This result is expected as most of these metabolites are signalling mediators which are produced locally. We also compared the variation of each metabolite due to the analytical variation (quality control samples) with the biological variation of that metabolite (in different biological samples) at the endogenous metabolite levels in the different tissues (Table S8). In almost all cases the procedural RSDs were significantly lower than the biological variation, comparable to the findings for other metabolites reported by Alves et al. [47].

**Fig. 5:**
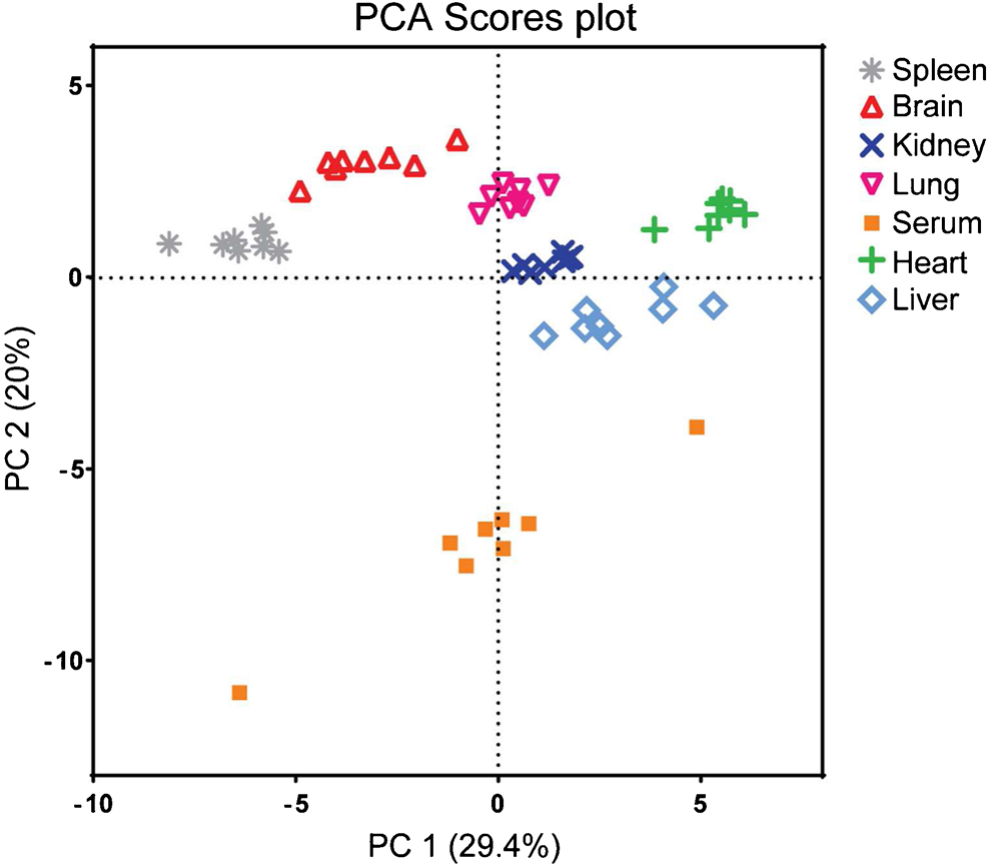
A principal component analysis (PCA) scores plot of the paired tissue and serum samples of eight healthy C57BL/6 mice, with complete differentiation visible between the six different tissues types and serum. PC principal component

Next we investigated the natural projection of the metabolite levels in the various tissues and serum samples using unsupervised multivariate PCA. Inspection of the PCA results revealed that the samples of different tissue types and the serum samples were completely separated, as shown in Fig. 5. The serum samples had the highest biological within sample type group variation, whereas the tissue groups clustered more closely together. The large variation present between the different serum samples emphasizes the function of serum as a circulatory system in the body, connecting the different tissues and organs, leading to a unique readout characteristic for the study subject. The different number of metabolites detected in the different tissues contributed to the complete PCA separation. Subsequently, we compiled a data set consisting of only the six tissues and only metabolites found in all tissues, and redid the PCAs (Fig. S5). Even after the data set had been reduced, clear differentiation between the six tissues was still observed, emphasizing the tissue-specific stress and inflammatory profile, and was not due to metabolites detected in only one or a few but not all tissues.

**Fig. 6:**
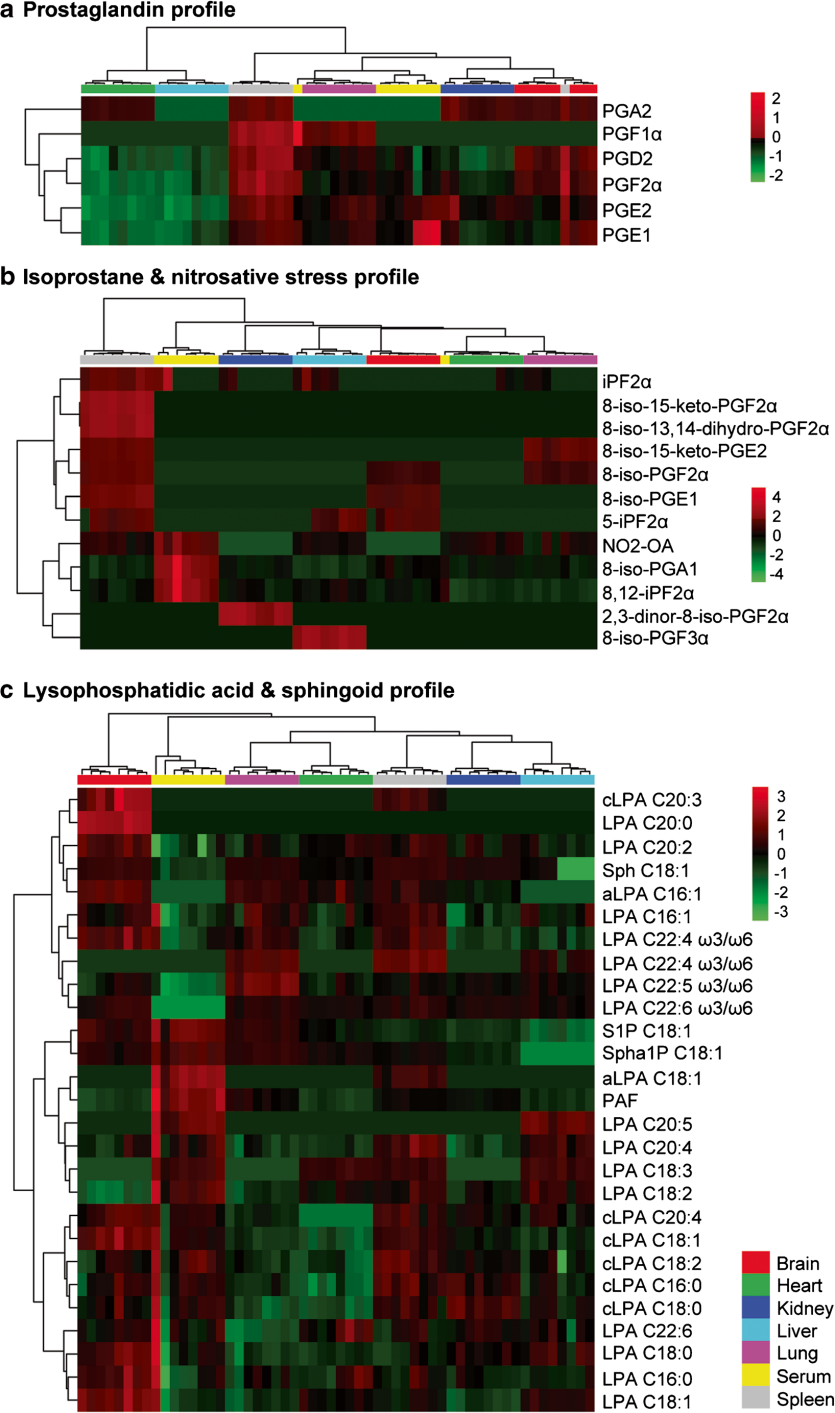
Heatmaps of all metabolites detected divided into prostaglandins (a), isoprostanes and nitro fatty acids (c) and lysophosphatidic acids and sphingoid bases (c) by Euclidean distance measure and the Ward clustering algorithm. aLPA alkyl lysophosphatidic acid, cLPA cyclic lysophosphatidic acid, iP isoprostane, LPA lysophosphatidic acid, PAF platelet-activating factor, PG prostaglandin, OA oleic acid, S1P sphingosine 1-phosphate, Sph sphingosine, Spha1P sphinganine 1-phosphate

To compare the different metabolite levels in the different tissue samples, heatmaps were used to visualize the individual measurements. The signalling lipid profile was split into the prostaglandins (Fig. 6, panel a), the isoprostanes and NO_2_-FAs (Fig. 6, panel b) and the LPAs and sphingoids (Fig.6, panel c). First, we investigated the prostaglandin profile (Fig. 6, panel a) and found that the spleen has the highest level of prostaglandins present. The brain also shows high prostaglandin levels even with the reduced extraction efficiency, with prostaglandin D_2_ (PGD_2_) and prostaglandin F_2α_ (PGF_2α_) being the dominant species. The heart and liver samples both have the lowest levels of prostaglandins detected. The kidney samples have high levels of especially PGE_2_ and PGA_2_, and the lung has high levels of prostaglandin F_1α_. The reasons for these unique prostaglandin profiles could be attributed to their signalling functioning and its relation to the specific tissue function. The spleen is an interface between the circulating blood and lymph systems critical for innate and adaptive immune responses, especially against bacterial and fungal infections. It is also essential in regulating erythrocyte level. The presence of high levels of prostaglandins in the spleen highlights the immunological importance of these lipid mediator species in orchestrating the differentiation, migration and response of leucocytes [48, 49]. High levels of PGE_2_ in the kidney relates to its homeostatic role in fluid metabolism and hemodynamic effects. PGE_2_ regulates blood flow in the kidney and sodium excretion, influencing blood pressure [50–52]. PGD_2_ has been identified as the main central nervous system prostaglandin in rats, functioning in a protective manner [53, 54]. The low levels of prostaglandins detected in the heart and liver possibly reflect the health status of the mice. Serum prostaglandin readouts were quite diverse, with some samples presenting especially high levels of prostaglandin E_1_ (and to a lesser extent PGE_2_), and other samples having much lower levels. Even though these mice present baseline levels, some physiological state cannot be excluded, and the differences found might be subtle, or important.

Next we zoomed in on the homeostatic stress-related lipid profile consisting of the isoprostanes and NO_2_-FAs (Fig. 6, panel b). The most characterized and described isoprostane, 8-iso-PGF_2α_, was detected only in brain, lung and spleen tissues, whereas the isoprostane 8,12-isoprostane F_2α_ IV (8,12-iPF_2α_ IV) was detected in all six tissues and serum, and might be a more sensitive readout for oxidative stress. The downstream metabolite of 8-iso-PGF_2α_, 2,3-dinor-8-iso-PGF_2α_, was strongly detected in serum and kidney tissue, revealing the metabolizing and excretion of oxidative stress markers via urine. Nitro-oleic acid was detected in all tissues and serum except for brain, heart and kidney. Fu et al. [55] reported that urine provides an excellent sample matrix for assessing the levels of NO_2_-FAs. Overall, the spleen presented the highest levels of oxidative stress markers. It is interesting to note that the different tissues all had different types of isoprostanes present. As lipid oxidation and nitration via ROS and RNS is a non-enzymatic, non-specific process, the homeostatic levels are possibly reflective of low-level stress and redox biology processes and that this is to some extent probably tissue function related.

Lastly, we focused on the LPAs and sphingoids (Fig. 6, panel c). The brain is a rich source of LPA as is the spleen. The highest levels of LPA have been found in brain tissues compared with the liver, lung and heart in rats [56]. The function of LPA in the spleen relates to its ability to induce chemokines in T cells, regulating their migration to secondary lymphoid tissues [57]. S1P and sphinganine 1-phosphate were detected at high levels in serum and at lower levels in tissue samples, whereas sphingosine and sphinganine had lower serum levels and higher tissue levels. This S1P gradient supports the vascular function of S1P, important in endothelial barrier integrity via its G-protein-coupled S1P receptor 1 [58, 59]. In tissues, the synthesis of S1P is dependent on sphingosine levels, and after synthesis, S1P is either excreted or metabolized. Even though the spleen is a rich source of blood, the signalling capacity of S1P in the spleen is of critical importance to the migration of B cells and T cells. Ramos-Perez et al. [60] found in an elegant study that the spleen has exquisitely tight regulation of S1P levels and that the level of S1P in the spleen was very low compared with that in the circulating plasma.

### Serum provides a non-tissue-specific homeostatic stress and inflammation readout

**Fig. 7:**
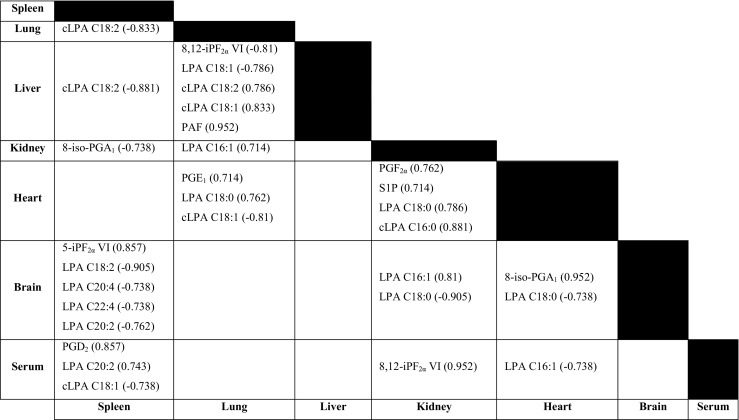
Spearman correlation analyses of paired tissue and serum samples. All correlations had *p* < 0.05, and the correlation coefficient is shown with its corresponding metabolite. cLPA cyclic lysophosphatidic acid, iP isoprostane, LPA lysophosphatidic acid, PAF platelet-activating factor, PG prostaglandin, S1P sphingosine 1-phosphate

As shown in the preceding sections, each tissue clearly has a distinct homeostatic metabolic stress and inflammatory profile primarily related to tissue function; therefore, it is interesting to determine how reflective serum is as a systemic readout of these different tissue profiles. Because of the relative ease and non-invasive nature of the collection protocols for most biofluids (serum, plasma and urine), they are frequently the sample material of choice in studies investigating health and disease, although a possible drawback of biofluids is that they represent a systemic readout of a highly dynamic system rather than allowing tissue-specific readouts. For the metabolites studied in this method, it is still rather unclear whether the serum profile reflects the physiology of the various tissues, as it is difficult to determine the origins of most metabolites in serum. It is generally believed that localized disease perturbations in the body are severe enough to spill over into the circulation, where they can be measured in a less invasive manner. This is also the fundamental principle in disease biomarker studies, where systemic circulating metabolites are sensitive and specific for a particular disease, acting as a biomarker (biosignature or disease fingerprint) [61, 62]. However, when biological investigations are being conducted into the pathogenic mechanism governing diseases, it is debatable whether we can draw correct conclusions using a systemic readout only. Because of the paired nature of the samples used, we performed Spearman correlation analyses between the same metabolites in the different tissues and serum samples, revealing an interesting picture resulting in both positive and negative correlations (Fig. 7).

Three metabolites correlated significantly between serum and spleen tissue, and one correlation was found for kidney and heart tissues; these were all different metabolites. Urade et al. [63] reported that the major source of endogenous PGD_2_ derived from a glutathione-dependent PGD_2_ synthase is produced by antigen-presenting cells, of which the spleen is a rich source under homeostasis, supporting this finding. The isoprostane 8,12-iPF_2α_ VI correlated strongly between serum and kidney tissue samples, suggesting the excretion of this metabolite via urine. Both positive and negative LPA correlations were observed between the different tissues, the interpretation of which remains unclear. Especially spleen and brain tissue indicated a strong correlation, with four unsaturated LPA species identified as significantly negatively correlated.

From the data taken collectively, few correlations were found between circulating serum and tissue metabolism when we investigated the baseline stress and inflammatory profiles of healthy mice. Furthermore, the subtle correlations could allude to organ regulation of signalling metabolites as well as intraorgan metabolic compartmentalization. It is quite well known that different organs are composed of different tissue subtypes. For example, the spleen consists of white and red pulp, with each having unique properties, functions and quite possibly also metabolism. Thus, when metabolic questions related to health and disease are being studied the choice of the proper sample material is of critical importance to accurately reflect the pathological condition investigated.

## Concluding discussion

Oxidative stress can be assessed with different techniques depending on the experimental design and sample material available. Currently the use of in vitro assays measuring hydrogen peroxide levels and glutathione ratios (also in vivo) is viewed as the most reliable approach in assessing the oxidative stress status. Newer technology such as Seahorse XF provides the opportunity to gain unique insights into mitochondrial functioning under different non-endogenous stressor conditions ideally suited for in vitro studies. However, the in vivo suitability of this live-cell analyses has some limitations when inadequate sample collection procedures have been followed. The application of the method presented in this article is applicable to both in vitro (cell lysate and medium) and in vivo (biofluids and tissues) studies. The non-specificity of free-radical attack makes the lipid peroxidation markers (isoprostanes) a generic readout for oxidative stress, which can be broadly applied. Furthermore, the inclusion of other signalling lipids adds to the understanding of inflammatory processes, and these mediators could potentially provide different readouts depending on the biological questions investigated. However, many questions remain about their function, in part because only a few tools can accurately measure their location specifically in vivo*.*

As oxidative stress and inflammation are central to many known diseases, the development of a method able to provide systemic and localized readouts is of the utmost importance. In addition, in some tissues and some diseases (cardiovascular), nitrosative stress is an important process. The method developed and validated in this work provides a stress readout based on the isoprostanes and NO_2_-FAs reflective of lipid peroxidation and nitration, as well as an inflammatory readout based on the prostaglandins, LPAs, cLPAs and sphingoids. Application of this method to biological questions related to health and disease will broaden our understanding of oxidative stress and inflammation at the metabolic level. The application of this metabolomics profiling method to healthy mice found that the systemic (serum profile) readout for stress and inflammation markers had little correlation to the localized readout of the six tissues tested under baseline conditions. However, we did identify specific serum metabolites correlating with levels in the spleen, heart and kidney as well as significant tissue–tissue metabolic correlations. In addition, each tissue type presented a unique homeostatic stress and inflammation profile. This might be due to the tightly controlled nature of these potent biological signalling lipids during homeostasis. In the event of a severe health perturbation, the reflective nature of this panel of metabolites in serum needs to be evaluated as the localized perturbation can spill over into a system readout, while also affecting other tissues and organs. Localized metabolomics studies have the potential to aid in biomarker discovery, elucidation of pathogenic mechanisms, prediction of disease severity, risk stratification and measurement of therapeutic intervention.

## Electronic supplementary material

ESM 1 (PDF 1.40 mb)
