# Supplementary material for: Development and application of a UHPLC–MS/MS metabolomics based comprehensive systemic and tissue-specific screening method for inflammatory, oxidative and nitrosative stress
Source: Anal Bioanal Chem. 2018 Mar 2;410(10):2551–68. doi: 10.1007/s00216-018-0912-2 (PMC5857282; doi:10.1007/s00216-018-0912-2)
Supplement: Supplementary file 1 — (PDF 1.40 mb) [file 216_2018_912_MOESM1_ESM.pdf]

Analytical and Bioanalytical Chemistry

Electronic Supplementary Material

Development and application of a UHPLC-MS/MS metabolomics based comprehensive systemic and tissue-specific screening method for inflammatory, oxidative and nitrosative stress

Johannes C. Schoeman, Amy C. Harms, Michel van Weeghel, Ruud Berger, Rob J. Vreeken, Thomas Hankemeier

Table S1 Target list with optimised MRM transitions and retention times for the identified metabolites, putative identified metabolites and ISTDs

Compound class	Compound name	Retention time (min)	Precursor ion> Product ion (m/z)	CE (V)	Dwell time (msec)	MS Polarity	Internal standard	Lipid Maps ID
	Identified metabolites based on standards							
Alkyl-lysophosphatidic acid *	aLPA C18:1	3.13	421.20>78.95	26	12	Neg	LPA C17:0	x
Cyclic-lysophosphatidic acid *	cLPA C18:1	3.70	417.20>281.00	17	12	Neg	cLPA C17:0	LMGP00000056
Isoprostane	2,3-dinor-11B-iso-PGF2α	3.12	325.00>145.20	20	20	Neg	(d4) PGF2α	LMFA03010011
Isoprostane	2,3-dinor-8-iso-PGF2α	2.89	325.10>237.20	12	20	Neg	(d4) 8-iso-PGF2α	LMFA03110010
Isoprostane	5-iPF2α VI	4.70	353.30>115.05	22	40	Neg	(d11) 5-iPF2α VI	LMFA03110010
Isoprostane	8,12-iPF2α VI	6.20	353.30>115.05	22	40	Neg	(d11) 8,12-iPF2α VI	
Isoprostane	8-iso-13,14-dihydro-PGF2α	4.98	353.30>183.10	25	20	Neg	(d4) 8-iso-PGF2α	
Isoprostane	8-iso-15-keto-PGE2	5.19	349.10>287.20	16	20	Neg	(d4) 8-iso-PGE2	LMFA03110009
Isoprostane	8-iso-15-keto-PGF2α	4.55	351.10>315.15	22	20	Neg	(d4) 8-iso-PGF2α	LMFA03110005
Isoprostane	8-iso-15-keto-PGF2b	4.40	351.10>315.15	22	20	Neg	(d4) 8-iso-PGF2α	
Isoprostane	8-iso-15-R-PGF2α	4.17	353.30>193.20	25	20	Neg	(d4) 8-iso-PGF2α	
Isoprostane	8-iso-PGA1	6.77	335.10>273.15	19	20	Neg	(d4) PGA2	LMFA03110008
Isoprostane	8-iso-PGA2	6.71	333.10>271.20	15	20	Neg	(d4) PGA2	
Isoprostane	8-iso-PGE1	5.18	353.30>317.20	15	20	Neg	(d4) PGE1	LMFA03110002
Isoprostane	8-iso-PGE2	4.97	351.10>271.15	17	20	Neg	(d4) 8-iso-PGE2	LMFA03110003
Isoprostane	8-iso-PGF1α	4.39	355.30>311.10	22	20	Neg	(d9) 8-iso-PGF1α	
Isoprostane	8-iso-PGF2α (15-F2t-IsoP)	4.37	353.30>193.20	25	20	Neg	(d4) 8-iso-PGF2α	LMFA03110001
Isoprostane	8-iso-PGF3α	3.52	351.10>307.15	19	20	Neg	(d4) 8-iso-PGF2α	LMFA03110007
Isoprostane	iPFa-IV	4.05	353.30>127.10	22	20	Neg	(d4) iPFa-VI	
Lysophosphatidic acid *	LPA C16:0	3.11	409.00>153.05	22	12	Neg	LPA C17:0	LMGP10050006
Lysophosphatidic acid *	LPA C18:0	3.40	437.30>153.05	24	12	Neg	LPA C17:0	LMGP10050005
Lysophosphatidic acid *	LPA C20:4	3.03	456.70>153.05	22	12	Neg	LPA C17:0	LMGP10050013
Sphingoid	Sph C18:1	8.03	300.10>252.30	-18	8	Pos	Sph C17:1	LMSP01010001
Sphingoid	Spha C18:0	8.18	302.50>60.05	-22	8	Pos	Spha C17:0	LMSP01020001
Sphingoid *	Spha1P C18:0	3.24	380.00>79.05	41	12	Neg	Spha1P C17:0	LMSP01050002
Sphingoid *	S1P C18 :1	3.12	378.00>79.05	25	12	Neg	S1P C17:1	LMSP01050001
Nitro-Fatty acid	NO2-LA (C18:2)	8.40	324.30>277.25	12	40	Neg	10-Nitrooleate-d17	LMFA01120001/2
Nitro-Fatty acid	NO2-OA (C18:1)	8.55	326.10>46.00	14	40	Neg	10-Nitrooleate-d17	LMFA01120003/4
Platelet activating factor *	PAF C16:0	4.66	524.20>125.10	-25	15	Pos	PAF C18:0-d4	LMGP01020046
Prostaglandins	13,14-dihydro-PGF2α	5.99	353.30>183.10	25	20	Neg	(d4) PGF2α	LMFA03010079
Prostaglandins	PGA1	7.08	335.10>273.15	19	20	Neg	(d4) PGA2	LMFA03010005
Prostaglandins	PGA2	6.82	333.10>271.20	15	20	Neg	(d4) PGA2	LMFA03010035
Prostaglandins	PGD2	5.42	351.10>271.15	17	20	Neg	(d4) PGD2	LMFA03010004
Prostaglandins	PGD3	4.40	349.10>269.20	17	20	Neg	(d4) PGD2	LMFA03010142
Prostaglandins	PGE1	5.55	353.30>317.20	15	20	Neg	(d4) PGE1	LMFA03010134
Prostaglandins	PGE2	5.22	351.10>271.15	17	20	Neg	(d9) PGE2	LMFA03010003
Prostaglandins	PGE3	4.25	349.10>269.20	17	20	Neg	(d9) PGE2	LMFA03010135

Prostaglandins	PGF1α	5.25	355.30>311.10	22	20	Neg	(d9) 8-iso-PGF1α	LMFA03010137
Prostaglandins	PGF2α	5.15	353.30>193.20	25	20	Neg	(d4) PGF2α	LMFA03010002
Prostaglandins	PGF3α	4.15	351.10>307.15	19	20	Neg	(d4) PGF2α	LMFA03010138
	**Putatively identified metabolites**							
Alkyl-lysophosphatidic acid *	aLPA C16:1	3.00	393.20>78.95	25	12	Neg	LPA C17:0	x
Cyclic-lysophosphatidic acid *	cLPA C16:0	3.62	390.90>255.00	20	12	Neg	cLPA C17:0	LMGP00000057
Cyclic-lysophosphatidic acid *	cLPA C18:0	3.93	418.90>283.00	20	12	Neg	cLPA C17:0	LMGP00000055
Cyclic-lysophosphatidic acid *	cLPA C18:2	3.49	414.90>279.00	20	12	Neg	cLPA C17:0	x
Cyclic-lysophosphatidic acid *	cLPA C20:3	3.63	440.90>305.00	20	12	Neg	cLPA C17:0	x
Cyclic-lysophosphatidic acid *	cLPA C20:4	3.48	438.90>303.00	20	12	Neg	cLPA C17:0	x
Lysophosphatidic acid *	LPA C14:0	2.85	381.20>153.05	22	12	Neg	LPA C17:0	LMGP10050007
Lysophosphatidic acid *	LPA C16:1	2.94	407.20>153.05	22	12	Neg	LPA C17:0	LMGP10050016
Lysophosphatidic acid *	LPA C18:1	3.13	435.20>153.05	22	12	Neg	LPA C17:0	LMGP10050008
Lysophosphatidic acid *	LPA C18:2	3.02	433.20>153.05	22	12	Neg	LPA C17:0	LMGP10050017
Lysophosphatidic acid *	LPA C18:3	2.91	431.20>153.05	22	12	Neg	LPA C17:0	LMGP10050023
Lysophosphatidic acid *	LPA C20:0	3.90	465.20>153.05	22	12	Neg	LPA C17:0	LMGP10050018
Lysophosphatidic acid *	LPA C20:1	3.50	463.20>153.05	22	12	Neg	LPA C17:0	LMGP10050026
Lysophosphatidic acid *	LPA C20:2	3.32	461.20>153.05	22	12	Neg	LPA C17:0	LMGP10050027
Lysophosphatidic acid *	LPA C20:3	3.08	459.20>153.05	22	12	Neg	LPA C17:0	LMGP10050028
Lysophosphatidic acid *	LPA C20:5	2.91	455.20>153.05	22	12	Neg	LPA C17:0	LMGP10050033
Lysophosphatidic acid *	LPA C22:4	3.19	485.20>153.05	22	12	Neg	LPA C17:0	LMGP10050020
Lysophosphatidic acid *	LPA C22:5	3.10	483.20>153.05	22	12	Neg	LPA C17:0	x
Lysophosphatidic acid *	LPA C22:6	2.99	481.20>153.05	22	12	Neg	LPA C17:0	LMGP10050019
Nitro-Fatty acid	NO2-aLA (C18:3)	8.25	322.10>46.05	12	12	Neg	10-Nitrooleate-d17	
	**Internal standards**							
Cyclic-lysophosphatidic acid *	cLPA C17:0	3.77	405.20>269.25	20	12	Neg	*na*	
Isoprostane	5-iPF2α-VI-d11	4.68	364.20>115.05	22	20	Neg	*na*	LMFA03110011
Isoprostane	8-iso-PGE2-d4	4.93	355.30>275.25	18	20	Neg	*na*	LMFA03010008
Isoprostane	8-iso-PGF1α-d9	4.36	364.20>320.25	23	20	Neg	*na*	
Isoprostane	8-iso-PGF2α-d4	4.35	357.30>197.15	20	20	Neg	*na*	
Isoprostane	iPF2α-VI-d4	4.70	357.20>114.90	22	20	Neg	*na*	
Isoprostane	8,12-iso-iPF2α-VI-d11	6.17	364.20>115.05	23	40	Neg	*na*	
Lysophosphatidic acid *	LPA C17:0	3.19	423.20>153.05	22	12	Neg	*na*	LMGP10050036
Sphingoid	Sph C17:1	7.82	285.90>238.30	-14	8	Pos	*na*	
Sphingoid	Spha C17:0	7.94	288.10>59.80	-18	8	Pos	*na*	
Sphingoid *	S1P C17:1	2.91	364.00>78.95	30	12	Neg	*na*	
Sphingoid *	Spha1P C17:0	3.01	366.00>79.05	28	12	Neg	*na*	
Nitro-Fatty acid	10-Nitrooleate-d17	8.53	343.10>46.05	15	20	Neg	*na*	
Platelet activating factor *	PAF C16:0-d4	4.65	527.70>125.00	-52	15	Pos	*na*	
Prostaglandins	PGD2-d4	5.40	355.30>275.25	18	20	Neg	*na*	LMFA03010007
Prostaglandins	PGF2α-d4	5.13	357.30>197.15	20	20	Neg	*na*	LMFA03010006
Prostaglandins	PGE2-d9	5.20	359.90>280.25	18	20	Neg	*na*	
Prostaglandins	PGE1-d4	5.53	357.30>321.20	15	20	Neg	*na*	
Prostaglandins	PGA2-d4	6.80	336.90>275.00	11	20	Neg	*na*	
Reference	Cuda	7.58 / 2.65*	339.30>214.25	24		Neg	*na*	

* Indicates that the metabolite is measured during the high pH run

Table S2 Provides an overview of the concentrations of the calibration solution and eventual spiked levels obtained during this study

Calibration level	Stock concentration (nM)	dilution factor based on C8 level	Calibration level when 20 µL is spiked into 150µL sample (nM)
**C8**	1304*/4000#	-	173/533
**C7**	1004/3080	0.77	133/410
**C6 (high)**	495/1520	0.38	66/202
**C5**	247/760	0.19	33/101
**C4 (Medium)**	104/320	0.08	13/42
**C3**	10/30.8	0.0077	1.3/4.1
**C2 (Low)**	2.5/7.6	0.0019	0.3/1.0
**C1**	0.75/2.3	0.00058	0.09/0.3
**C0**	0/0	0	0/0

* Isoprostanes, prostanoids and nitro-fatty acids concentrations

Lysophosphatidic acid and sphingoid base standards concentrations

Table S3 Lysophosphatidic acid and cyclic-lysophosphatidic acid species target list

Lysophosphatidic acid targets	Dehydrated parent (M-H)$^-$ (m/z)	Fragments		Retention time (min)#	Cyclic-lysophosphatidic acid targets	Dehydrated Parent (M-H)$^-$ (m/z)	Fragments		Retention time (min)#
		Dehydrated glycerol phosphate (m/z)	Phosphate (m/z)				Fatty acid (m/z)	Phosphate (m/z)	
LPA C14:0	381.2	152.9	78.9	2.85	cLPA C14:0	363.2	227.2	78.9	ND
**LPA C16:0 ****	409.2	152.9	78.9	3.11	cLPA C16:0	391.2	255.2	78.9	3.62
LPA C16:1	407.2	152.9	78.9	2.94	cLPA C16:1	389.2	253.2	78.9	ND
**LPA C17:0 ****	423.9	152.9	78.9	3.19	**cLPA C17:0 ****	405.2	269.2	78.9	3.77
**LPA C18:0 ****	437.2	152.9	78.9	3.4	cLPA C18:0	419.2	283.2	78.9	3.93
LPA C18:1	435.2	152.9	78.9	3.13	**cLPA C18:1 ****	417.2	281.2	78.9	3.7
LPA C18:2	433.2	152.9	78.9	3.02	cLPA C18:2	415.2	279.2	78.9	3.49
LPA C18:3	431.2	152.9	78.9	2.91	cLPA C18:3	413.2	277.2	78.9	ND
LPA C18:4	429.2	152.9	78.9	ND	cLPA C18:4	411.2	275.2	78.9	ND
LPA C20:0	465.2	152.9	78.9	3.9	cLPA C20:0	447.2	311.2	78.9	ND
LPA C20:1	463.2	152.9	78.9	3.5	cLPA C20:1	445.2	309.2	78.9	ND
LPA C20:2	461.2	152.9	78.9	3.32	cLPA C20:2	443.2	307.2	78.9	ND
LPA C20:3	459.2	152.9	78.9	3.08	cLPA C20:3	441.2	305.2	78.9	3.63
**LPA C20:4 ****	457.2	152.9	78.9	3.03	cLPA C20:4	439.2	303.2	78.9	3.48
LPA C20:5	455.2	152.9	78.9	2.91	cLPA C20:5	437.2	301.2	78.9	ND
LPA C22:0	493.2	152.9	78.9	ND	cLPA C22:0	475.2	339.2	78.9	ND
LPA C22:1	491.2	152.9	78.9	ND	cLPA C22:1	473.2	337.2	78.9	ND
LPA C22:2	489.2	152.9	78.9	ND	cLPA C22:2	471.2	335.2	78.9	ND
LPA C22:4	485.2	152.9	78.9	3.19	cLPA C22:4	467.2	333.2	78.9	ND
LPA C22:5	483.2	152.9	78.9	3.10	cLPA C22:5	465.2	331.2	78.9	ND
LPA C22:6	481.2	152.9	78.9	2.99	cLPA C22:6	463.2	329.2	78.9	ND

** Available Commercial standards which was used to define column retention and elution sequence.

ND – Not Detected

Retention time based on optimized method.

Table S4 An overview of the conditions tested and each class's response in the selected setup

pH conditions	Low pH (<3.4)		High pH (10.3)<br>(* Addition of salt)	
Mobile phase B	Methanol + 0.1 % AA	Acetonitrile + 0.1 % AA	Methanol + 0.1 % NH_4OH	Acetonitrile+ 0.1 % NH_4OH
Mobile phase A	H_2O + 0.1 % AA	H_2O + 0.1 % AA	H_2O + 0.0625 % NH_4OH<br>* H_2O + 5mM NH_4CH_3COO + 0.0625 % NH_4OH	H_2O + 0.0625 % NH_4OH<br>* H_2O + 5mM NH_4CH_3COO + 0.0625 % NH_4OH
**Isoprostanes**	Isomer separation problems	*Good peak shape*	Good<br>*Matrix effects	Elutes too early < 20 % B<br>*Elutes too early < 20 % B
**Prostaglandins**	Isomer separation problems	*Good peak shape*	Good<br>*Matrix effects	Elutes too early < 20 % B<br>*Elutes too early < 20 % B
**Nitro-fatty acids**	*Good peak shape*	*Good peak shape*	*Good peak shape*<br>**Good peak shape*	*Good peak shape*<br>**Good peak shape*
**LPAs & aLPAs**	Peak tailing<br>Carry over > 20%	Peak tailing<br>Carry over > 20%	*OK peak shape*<br>Carry over > 5 %<br>**Carry over < 3%*	*OK peak shape*<br>Carry over > 5 %<br>**Carry over < 3%*
**cLPAs**	*Good peak shape*	*Good peak shape*	*Good peak shape*<br>**Good peak shape*	*Good peak shape*<br>**Good peak shape*
**S1P & Spha1P**	Peak tailing<br>Carry over > 20%	Peak tailing<br>Carry over > 20%	*OK peak shape*<br>Carry over > 5 %<br>**Carry over < 2%*	*OK peak shape*<br>Carry over > 5 %<br>**Carry over < 2%*
**Sph & Spha**	*Good peak shape*	Peak tailing	Good peak shape<br>Carry over > 5 %<br>* Carry over > 5 %<br>Bad Ion suppression due to LPC/PAF co-elution	Peak tailing<br>Carry over > 5 %<br>* Carry over > 5 %
**PAF**	Good peak shape but co-eluting with LPC18:0	Good peak shape but co-eluting with LPC *18:0*	*Good peak shape but co-eluting with LPC *18:0*	**Good peak & separating from LPC 18:0. The separation is sensitive to the column lifespan, and column overloading.*

Green/Italics - Good chromatographic performance; - No shading - Chromatographic issues as indicated; AA – Acetic acid

Table S5 Methodology validation recoveries and matrix effects in serum

		**% Recovery (Matrix)**					**Matrix effect**		
		**I4 - Day 1**	**I4 - Day 2**	**I4 - Day 3**	**Average**	**RSD**	**I2-low**	**I4-medium**	**I6-high**
Lysophosphatidic acid	LPA C17:0	101.4	78.5	96.5	92.1	13.0	1.03	0.72	0.45
	cLPA C17:0	95.8	112.1	106.9	104.9	7.9	1.53	2.02	0.97
Sphingoids	Sph C17:1	101.1	87.0	98.6	95.6	7.9	0.94	0.65	0.38
	S1P C17:1	111.3	94.8	101.7	102.6	8.0	0.89	0.78	0.90
	Spha C17:0	106.4	92.8	102.1	100.4	6.9	0.73	0.67	0.59
	Spha1P C17:0	105.3	109.7	122.2	112.4	7.8	1.28	1.10	0.86
Nitro Fatty acids	d17 10-O2-OA	94.1	106.2	102.3	100.9	6.1	0.25	0.26	0.34
Prostaglandins	d9-PGE2	91.6	93.7	97.4	94.2	3.1	1.16	1.13	0.81
	d4-PGE1	93.6	95.7	96.7	95.3	1.6	1.39	1.16	0.84
	d4-PGD2	95.1	94.0	95.8	95.0	0.9	1.11	1.13	0.79
	d4-PGF2α	93.6	97.3	95.5	95.5	1.9	1.00	1.11	0.80
	d4-PGA2	96.0	98.4	96.3	96.9	1.3	0.94	1.08	0.78
Isoprostanes	d9-8-iso-PGF1α	106.5	100.2	97.7	101.5	4.4	1.30	1.35	0.96
	d4-8-iso-PGF2α	100.0	97.1	97.5	98.2	1.5	1.26	1.33	0.93
	d4-8-iso-PGE2	95.9	95.8	96.6	96.1	0.4	1.30	1.30	0.91
	d11- + 5-iPF2α VI	97.5	90.6	93.5	93.9	3.7	1.18	1.16	0.81
	d4-iPF2α VI	101.1	93.6	94.5	96.4	4.2	0.74	0.74	0.58
	d11-8,12-iPF2α VI	96.3	92.8	92.9	94.0	2.1	0.75	0.71	0.54

Table S6 Six different tissue validation recoveries and matrix effect results

		% Recoveries						Matrix effect *					
		**Kidney**	**Spleen**	**Liver**	**Lung**	**Heart**	**Brain**	**Kidney**	**Spleen**	**Liver**	**Lung**	**Heart**	**Brain**
Lysophosphatidic acid	LPA C17:0	78.5	97	70.8	75.7	105.2	52.6	0.63	0.78	0.80	0.49	0.65	0.47
	cLPA C17:0	89.2	72.1	100.8	91.4	98.9	60.8	0.54	0.66	0.53	0.42	0.87	0.31
Sphingoids	Sph C17:1	112.5	96.6	103.2	103.5	86.6	81.3	0.95	0.94	1.22	0.64	0.61	0.43
	S1P C17:1	90.5	110.4	83	74.6	109.2	69.5	0.55	0.67	0.67	0.46	0.70	0.40
	Spha C17:0	109.0	102.7	101.2	137.9	121.0	71.5	0.37	0.42	0.40	0.27	0.37	0.14
Nitro Fatty acids	d17 10-NO2-OA	43.0	49.9	53.9	37.7	*ND*	37.8	0.40	0.29	0.29	0.26	0.43	0.28
Prostaglandins	d9-PGE2	121.3	106.3	80.7	95.9	88.8	61.0	1.04	1.07	0.96	0.68	1.00	0.58
	d4-PGE1	119.6	102.7	85.4	96.5	84.1	59.8	1.01	1.04	0.67	0.66	0.99	0.58
	d4-PGD2	113.0	95.7	75.6	94.1	85.3	57.7	1.04	1.07	0.92	0.73	1.02	0.61
	d4-PGF2α	113.7	99.9	75.3	96.4	84.4	57.7	1.00	1.02	0.75	0.65	0.98	0.53
	d4-PGA2	60.4	46.2	11.2	30.3	48.3	29.3	0.57	0.48	0.14	0.24	0.56	0.28
Isoprostanes	d9-8-iso-PGF1α	125.9	121.0	70.4	99.1	95.7	53.9	1.01	1.00	0.79	0.64	0.94	0.48
	d4-8-iso-PGF2α	118.4	105.5	65.9	102.0	87.9	55.7	0.98	1.00	0.79	0.65	0.98	0.52
	d4-8-iso-PGE2	111.1	97.7	62.4	92.3	83.5	57.8	1.01	1.04	0.56	0.66	0.96	0.56
	d11- + 5-iPF2α VI	128.6	93.9	80.0	105.7	94.5	54.7	1.23	1.11	0.93	0.78	1.07	0.62
	d4-iPF2α VI	119.9	97.2	77.1	99.5	94.1	56.5	1.27	1.16	0.94	0.82	1.10	0.66
	d11-8,12-iPF2α VI	110.0	90.0	94.7	102.2	88.9	58.5	1.23	1.13	1.22	0.80	1.06	0.70

ND: Not detected

* with 1 equal to no matrix effect

Table S7 Intraday precision RSD values of the metabolites spiked to the different tissues

		Tissue Intraday precision (% RSD, n = 4)					
**Class**	**Metabolites**	**Spleen**	**Lung**	**Liver**	**Kidney**	**Heart**	**Brain**
**Isoprostanes**	2,3-dinor-8-iso-PGF2α	5.8	2.9	11.9	5.3	4.5	9.1
	8-iso-PGF3α	4.7	7.3	13.5	2.0	5.5	8.9
	2,3-dinor-11b-$PGF_{2\alpha}$	2.8	4.1	19.2	2.7	1.9	7.5
	8-iso-15-keto-PGF2b	4.2	6.5	10.3	2.4	4.3	5.4
	8-iso-15-keto-PGE2	4.5	9.3	10.9	4.6	2.1	4.9
	8-iso-15-keto-PGF2α	3.9	9.6	11.2	3.8	4.2	5.2
	iPF2α	3.2	2.1	5.5	2.02	1.9	6.0
	8-iso-15(R)-PGF2α	4.6	3.5	18.2	0.6	2.8	5.2
	8-iso-PGF1α	1.6	3.9	20.6	8.0	2.2	3.3
	8-iso-13,14-dihydro-PGF2α	2.6	3.7	16.3	5.6	5.5	6.6
	8-iso-PGF2α	3.8	5.6	11.5	1.8	3.3	7.5
	8-iso-PGE2	3.1	3.5	4.2	2.7	2.2	3.6
	8-iso-PGE1	3.1	6.4	9.3	6.5	0.8	1.7
	5 iPF2α IV	3.3	2.0	16.2	3.1	3.1	5.3
	8-iso-PGA2	5.8	8.4	27.9	8.6	7.5	16.2
	8-iso-PGA1	7.0	5.4	10.7	9.4	6.4	14.8
	8,12-iPF2α VI	3.3	5.9	24.4	9.8	5.2	35.9
**Lysophosphatidic acids**	LPA C20:4	36.2	32.9	57.3	56.8	28.5	42.4
	LPA C16:0	32.7	19.2	16.0	46.7	41.1	33.9
	aLPA C18:1	35.4	35.4	17.9	13.6	21.0	15.7
	LPA C18:0	25.1	14.1	24.8	19.4	22.9	4.4
	cLPA C18:1	5.8	28.1	10.9	15.9	29.7	9.2
**Sphingoids**	Sph C18:1	12.5	7.4	14.5	10.1	6.2	7.6
	Spha C18:0	25.1	27.2	26.4	29.9	20.6	25.6
	S1P C18:1	17.0	7.9	11.2	16.1	24.2	10.0
**Nitro-Fatty acids**	NO2-OA	3.7	15.9	4.3	9.3	13.6	7.4
	NO2-LA	10.4	31.5	15.1	14.7	13.3	11.6
**Prostaglandins**	PGE3	9.5	6.8	4.4	13.3	6.6	9.4
	PGD3	4.5	4.5	27.0	7.2	1.6	6.3
	PGF3α	3.6	3.0	10.8	3.4	1.0	2.0
	PGE2	6.8	6.2	14.4	6.4	5.4	8.6
	PGE1	2.4	3.6	4.3	6.7	1.2	2.0
	PGD2	6.7	5.2	3.4	2.1	2.8	7.3
	PGF1α	1.4	5.4	15.0	8.2	2.1	5.1
	PGF2α	5.2	5.5	3.4	3.0	2.4	4.2
	13,14-dihydro-PGF2α	2.0	6.5	6.0	2.9	1.2	3.5
	PGA2	1.8	1.9	5.1	2.5	6.3	7.8
	PGA1	2.4	2.5	5.5	3.5	5.5	7.7

Table S8 Comparison of the precision of metabolites (averaged per class) determined for technical replicates and biological replicates. For a simplified overview we averaged the RSDs of the metabolites detected within a class in the different tissues

	Tissue types											
	Spleen		Lung		Liver		Kidney		Heart		Brain	
**Metabolite class response**	**Procedure % RSD (n = 12)**	**Biological % RSD (n = 8)**	**Procedure % RSD (n = 12)**	**Biological % RSD (n = 8)**	**Procedure % RSD (n = 12)**	**Biological % RSD (n = 8)**	**Procedure % RSD (n = 12)**	**Biological % RSD (n = 8)**	**Procedure % RSD (n = 12)**	**Biological % RSD (n = 8)**	**Procedure % RSD (n = 12)**	**Biological % RSD (n = 8)**
**Isoprostanes**	11.4	30.9	21.8	29.7	15.4	53.0	22.9	33.1	18.3	34.3	32.1	30.2
**Prostaglandins**	16.5	36.7	15.5	36.8	17.6	46.1	18.8	76.0	25.9	37.8	22.8	39.3
**Nitro-fatty acids**	26.7	44.5	45.0	91.4	34.7	19.1	*ND*	*ND*	28.9	33.1	*ND*	*ND*
**Sphingoids**	30.7	32.7	19.1	25.0	18.7	41.5	19.8	27.6	18.9	28.7	25.0	41.6
**LPA**	30.6	41.9	36.5	41.0	40.3	39.1	31.3	33.6	30.2	40.8	31.5	44.2
**aLPA**	48.0	46.2	15.8	27.7	*ND*	*ND*	22.2	25.0	32.3	120.5	18.4	28.0
**cLPA**	32.3	38.9	28.8	35.1	34.4	47.9	19.2	30.1	36.3	46.8	26.8	64.2

ND - Not detected

LPA - Lysophosphatidic acids, aLPA - Alkyl-lysophosphatidic acids; cLPA - Cyclic-lysophosphatidic acids

Figures

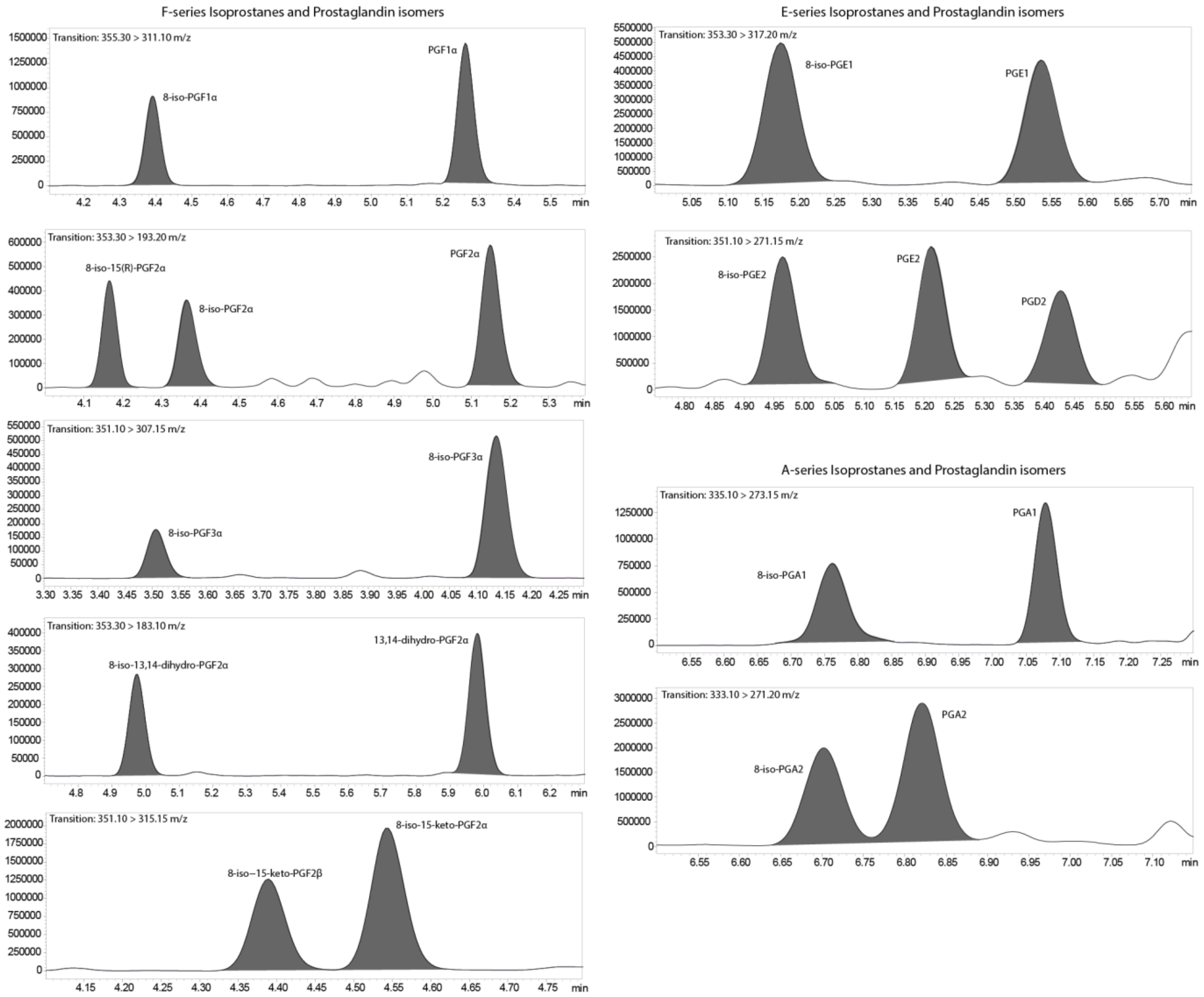

Fig. S1 Chromatographic resolution of the different prostaglandins and isoprostanes isomers

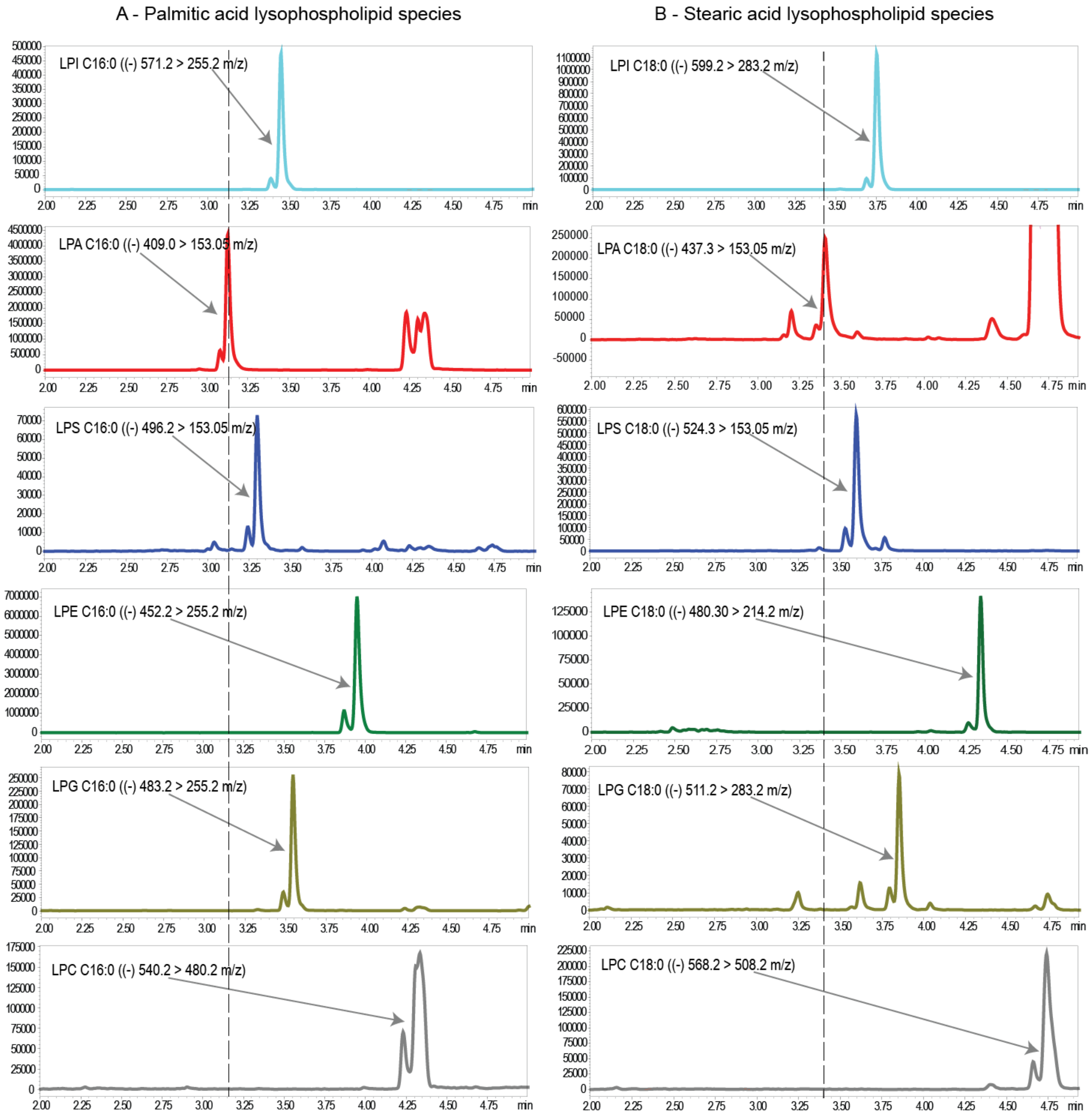

Fig. S2 A chromatographic overview of (a), palmitic acid (C16:0) and (b), stearic acid (C18:0) lysophospholipid species eluting during the high pH chromatographic run. LPA elutes first for both acyl lengths followed by LPS, LPI, LPG, LPE and lastly LPC. The dashed lines indicate the retention time of the two LPA species, and no co-elution is observed for the different lysophospholipid species of the same length. LPI – Lysophosphatidylinositol; LPA – Lysophosphatidic acid; LPS – Lysophosphatidylserine; LPE – Lysophosphatidylethanolamine; LPG – Lysophosphatidylglycerol; LPC – Lysophosphatidylcholine

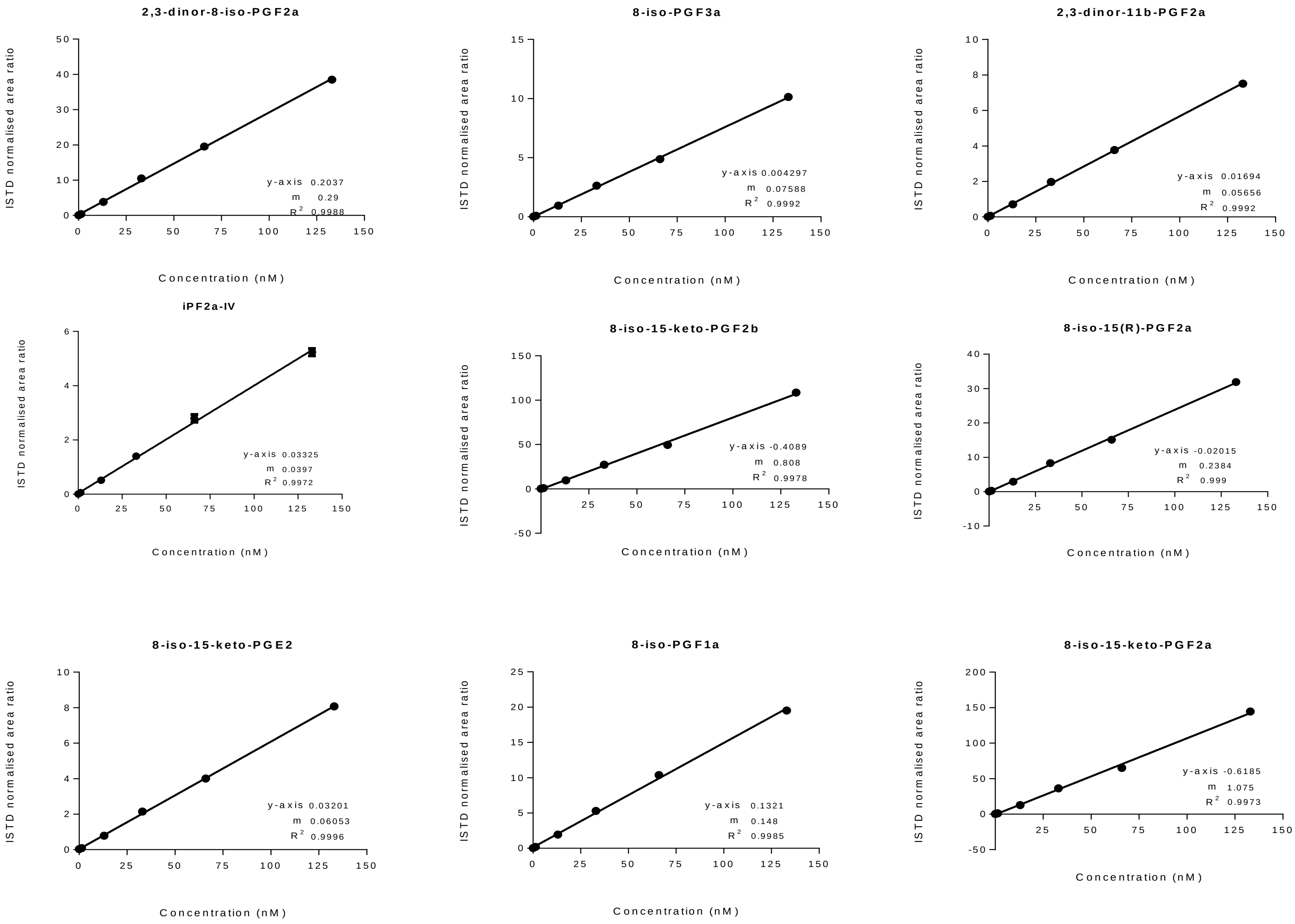

Fig. S3a Academic calibration curves for the isoprostanes, prostaglandin and nitro fatty acid standards

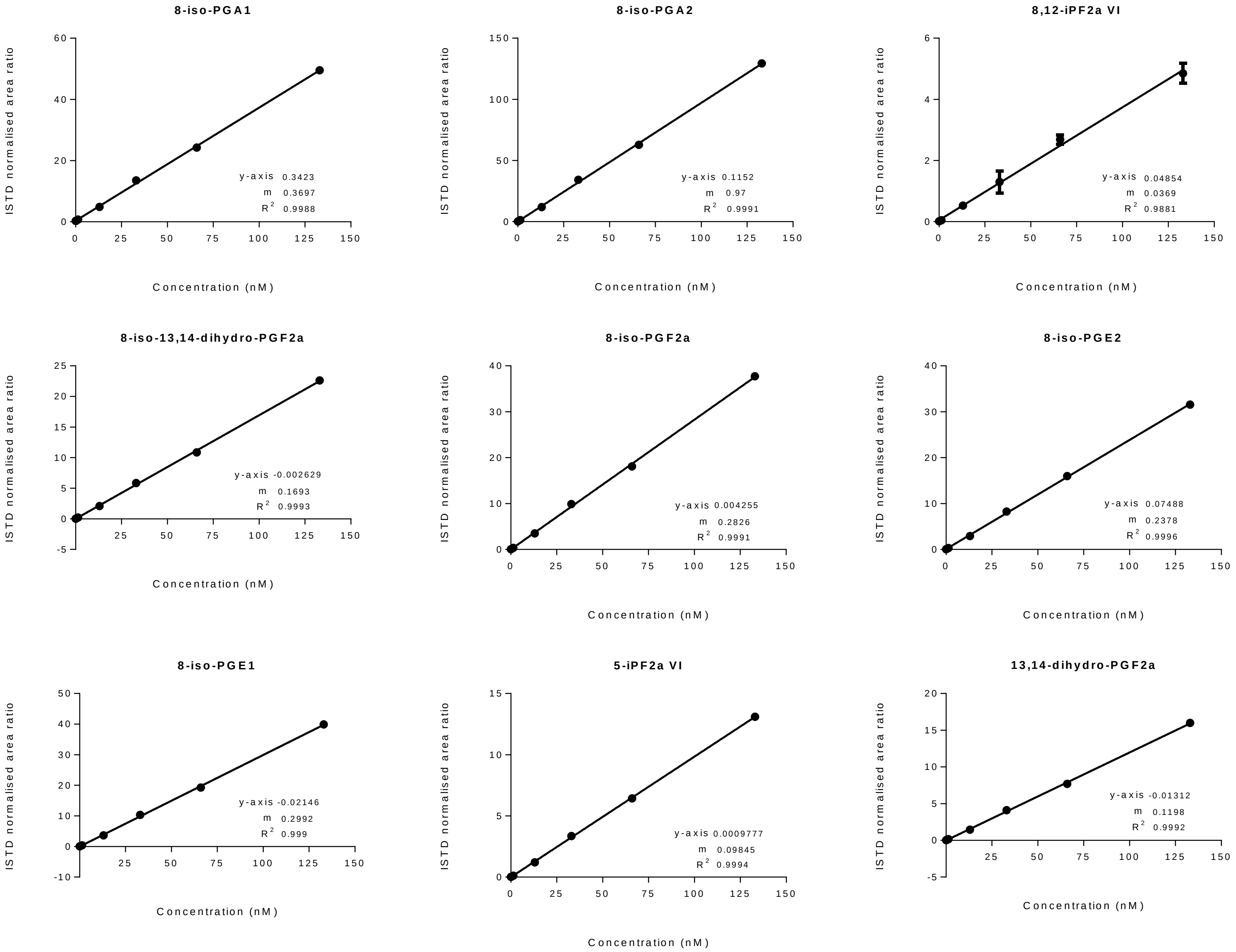

Fig. S3b Academic calibration curves for the isoprostanes, prostaglandin and nitro fatty acid standards

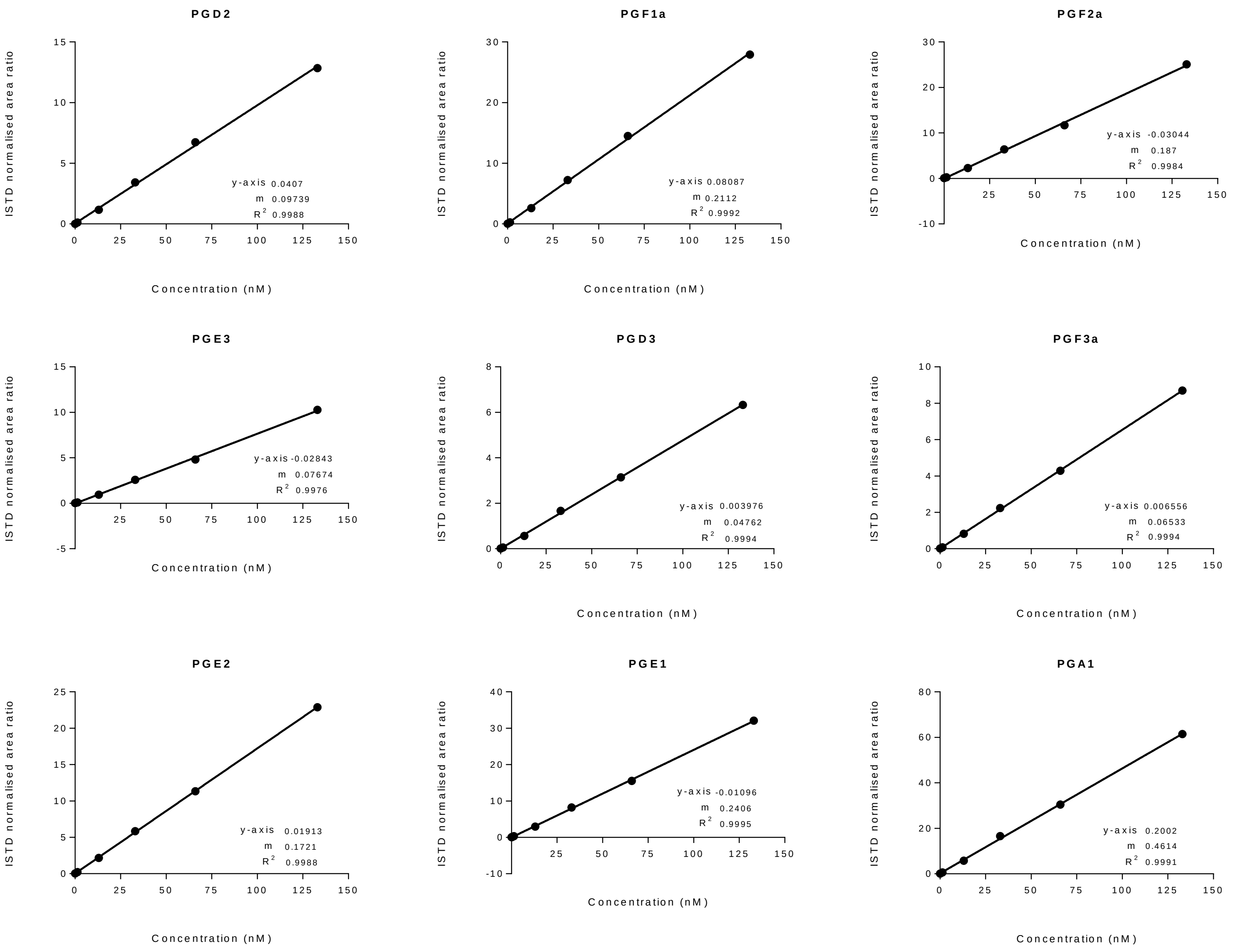

Fig. S3c Academic calibration curves for the isoprostanes, prostaglandin and nitro fatty acid standards

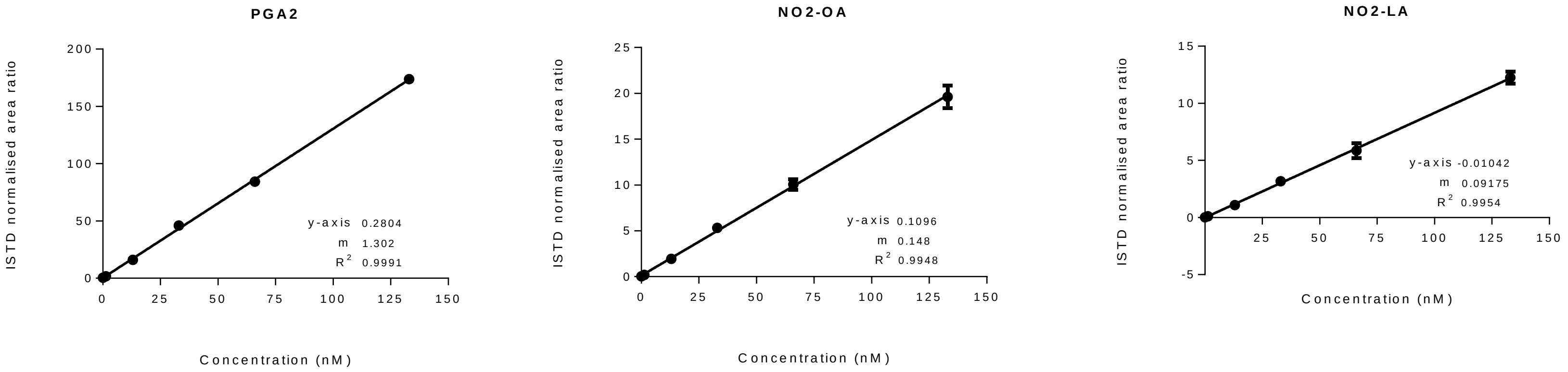

Fig. S3d Academic calibration curves for the isoprostanes, prostaglandin and nitro fatty acid standards

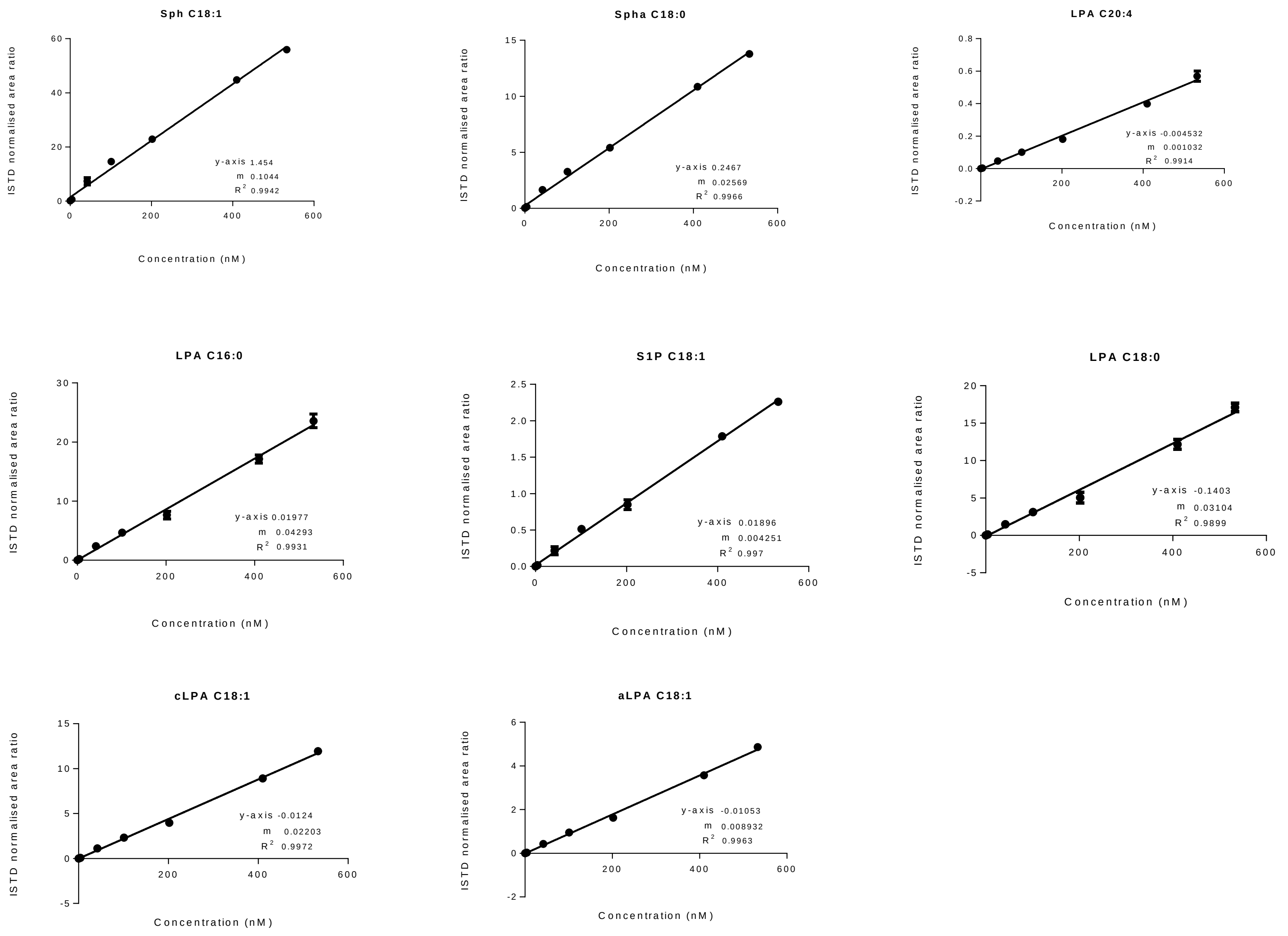

Fig. S3e Academic calibration curves for the sphingoid bases and the different lysophosphatidic acid

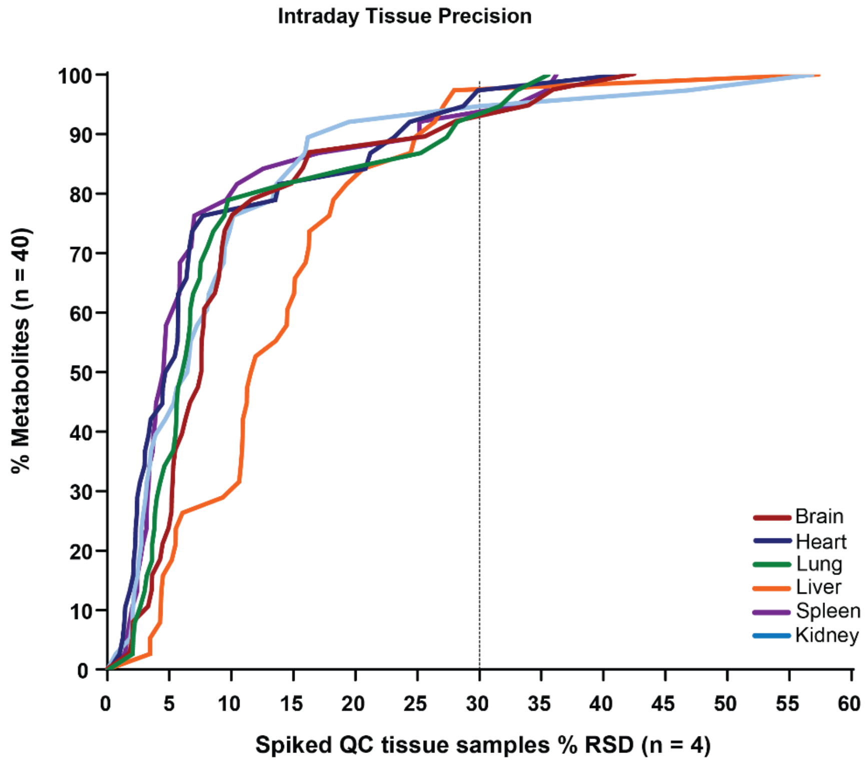

Fig. S4 Intraday precision in six different tissues. Precision was determined using the RSD (n=4) of C4 spiked standards into the different tissue matrixes

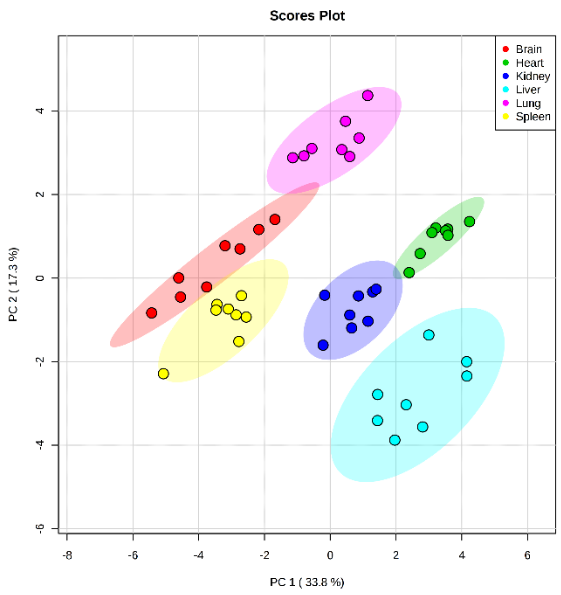

Fig. S5 Principal component analyses (PCA) Scores plot of the six different tissues investigated, using a reduced dataset containing only uniformly detected metabolites with all tissues.
